# Incorporation of polymerizable linkers into aptamers for high-affinity nanoscale molecularly imprinted polymer hybrids: analysis of positional selectivity†

**DOI:** 10.1039/d4tb02475c

**Published:** 2025-03-11

**Authors:** Mark V. Sullivan, Francia Allabush, Paula M. Mendes, James H. R. Tucker, Nicholas W. Turner

**Affiliations:** a Department of Chemistry, University of Sheffield Sheffield S3 7HF UK nicholas.turner@sheffield.ac.uk; b School of Chemical Engineering, University of Birmingham Edgbaston Birmingham B15 2TT UK; c School of Chemistry, University of Birmingham Edgbaston Birmingham B15 2TT UK

## Abstract

Aptamers are short single strand nucleic acid sequences that exhibit high-affinity molecular recognition towards non nucleic acid targets. They offer many benefits over antibodies, but still suffer from variable affinities and stability issues. Recently, aptamers have been incorporated as functional recognition agents into molecularly imprinted polymers, a competing recognition technology, to create hybrid materials, AptaMIPs, that exhibit the benefits of both classes. Specifically, this process can increase target affinity while preventing aptamer degradation. For the first time, using a lysozyme aptamer as an exemplar, we have undertaken a systematic and fundamental study to identify the optimal number and location of polymer connection points on an aptameric sequence for boosting AptaMIP target affinity and selectivity creating high affinity recognition elements. Clear patterns have emerged showing “fixing” throughout the molecule is required, but only in particular regions of the sequence. The results suggest that conformationally flexible regions within the polymer-bound aptameric sequence are detrimental to strong target binding, supporting the hypothesis that a successful imprinting process must lock the aptamer into its ideal binding conformation to achieve observable marked improvement in recognition. Conversely, too much flexibility in the embedded oligo (demonstrated through limited binding points) leads to poor performance. These findings offer a clear direction for development of aptamer–polymer hybrids. We also demonstrate the effectiveness of the developed materials in sensitive detection of the template using surface plasmon resonance, through improved quality of the recognition element.

## Abstract 2

Position, and number, of linkers between aptamer and polymer is critical for the performance of aptamer-molecularly imprinted polymer hybrids.

## Graphical abstract

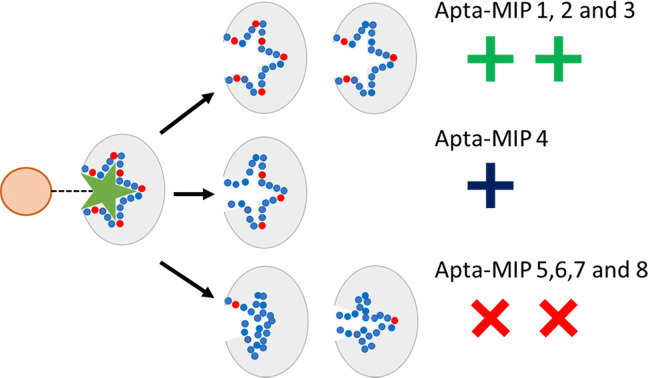

## 1. Introduction

Aptamers are short single strand nucleic acid sequences that exhibit high-affinity molecular recognition towards a target molecule, like the interaction observed by an antibody. Since the initial demonstrations of the technology^1,2^ and the subsequent development of SELEX (Systematic Evolution of Ligands by EXponential enrichment), they have become a significant player in the field of sensing, therapeutics, labelling, and other areas where targeted nanoscale molecular recognition is required.^3^

Today, aptamers exhibit high thermal/salt stability, excellent scalability in production with the ability to be generated for almost any epitope, even nonimmunogenic targets – providing tailored specificity and affinity. They also have a benefit of not relying on animal models for production, supporting the principles of 3Rs (replacement, reduction and refinement).^4^ Despite these significant benefits over antibodies, several challenges still need to be addressed including the wide range of observed affinities – aptamers exhibit equilibrium dissociation constants (*K*_D_'s) between millimolar and picomolar – whereas antibodies are less varied; potential toxicity; drug load capacity; and stability within the biological environment (exposure to nucleases *etc.*).^4,5^ Further questions exist around materials used to deliver aptamers, specifically around toxicity, biodegradation and accumulation.^6^

Specifically within the biosensor field, where aptamers are often demonstrated as effective selective binders for analytes, questions exist around stability, specificity, sensitivity, non-specific absorption of competing analytes under the required conditions, be it clinical or environmental samples.^6^ Naturally over the intervening 30+ years since discovery, a significant amount of research has occurred to expand the capabilities and enhance the properties of aptamers to improve recognition, stability, synthetic strategies and application.^3^ Given the depth of understanding around nucleic acid chemistry a wide variety of strategies have been explored to improve performance.^7,8^

The perceived challenges that exist for aptamers are also observed in another molecular recognition technique – that of molecular imprinting. This is a simple concept to visualise. A target template interacts with multiple monomers to form a reversible intermolecular complex, which is in turn entrapped within a polymeric scaffold using a suitable crosslinker (*via* a controlled polymerisation reaction). Once the final material is produced, the template is removed leaving behind a binding site in the polymer, one that is sterically and chemically complementary to the original template.^9^

While there are multiple “moving parts” involved in making a molecularly imprinted polymer (MIP), including selection of the cross-linker, initiator, reaction conditions, polymer format *etc.*, arguably the key component is that of monomer selection, which governs the type and number of interactions with the target template. We can easily visualise the principle of complementary functional groups in this interaction – for example, a secondary amine forming a hydrogen bond with a carboxylic acid group – but the actuality of the process is more complicated. The formation of the template–monomer complex is under thermodynamic control and this initial interaction, governed by ratios of monomer to template, is vital to final function and performance.^10,11^

Many strategies have been explored to improve monomer selection such as modelling and rational design,^10,12–14^ predetermined macromonomers such as cyclodextrins, calixarenes or crown ethers^15–18^ or structural pairing,^19–21^ each having a greater or lesser effect on performance. Beyond these materials, a strategy using nucleic acids has, in the past ten or so years, become an option through utilising the recognition granted by an aptamer as the “monomer”, essentially creating hybrid materials.^22^ This idea, using an aptamer within a polymer scaffold is an interesting one. Obviously, from a standpoint of molecular imprinting the potential to improve recognition is exciting, but if we consider this from a nucleic acid perspective there is significant potential in modifying sequences to allow them to interact covalently with polymers – a vast and exploitative field.

Spivak demonstrated a method in which aptamers selective for thrombin were embedded into hydrogels *via* a 5′-acrydite linker, doubling the recognition performance against their controls.^23^ Estrela and Bowen pioneered a “double-recognition” approach,^24^ using the idea of attaching an aptamer to a surface through suitable chemistry (*e.g.* 3′ thiol modification to gold), introducing the template, and then polymerising around the pairing, essentially using the aptamer for recognition and then building a binding pocket around the immobilised template. This has been copied in many other studies.^25–27^ This is ideal for sensor applications as a surface is used directly, however for other uses (such as therapeutics) this is self-limiting due to the need of a surface. Liu applied this idea to create nanoparticles, by attaching the aptamer to up-conversion (signal enhancing) nanoparticles as a core around which a shell of polymer was formed.^28^ This work demonstrated good results in the development of bioassays for antibiotics.

Despite, the successes of this overall method, questions remain on the environmental stability of the aptamer, and potential conformational changes of the aptamer within the binding pocket. This could limit recognition as the aptamer may form different 3D structures in the environment of the binding pocket, away from the optimal binding conformation, under the influence of the surrounding polymer. While these double-recognition systems offer interesting analytical data, only the Spivak study considers using the aptamer as a true functional monomer (*i.e.* part of the formal polymer matrix).

**Fig. 1:**
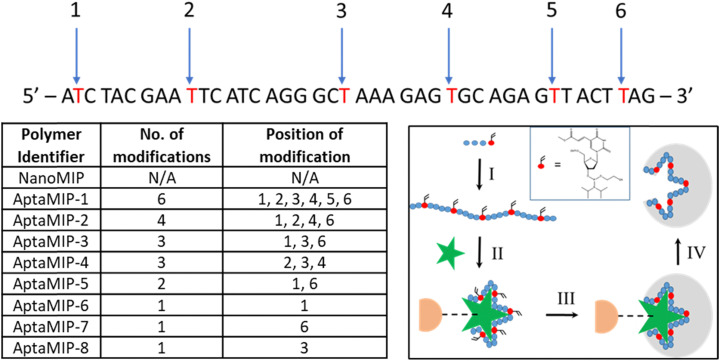
Modified aptamer sequences synthesised highlighting the position and number of modifications, and corresponding AptaMIP identifier codes. Inset: Schematic representation of the synthesis of the AptaMIP nanoparticles. Red circle indicates the modified polymerizable base (carboxy-dT-CE phosphonamidite). Orange circle represents the solid phase (silica). Green star representative of template. (I) Synthesis of modified aptamer sequence. (II) Complexation of aptamer with protein target attached to inert solid phase. (III) Addition of polymer scaffold components and polymerization reaction; (IV) thermal release of nanoparticle bearing aptamer sequence. Note: a positive control nanoMIP was made using the same solid-phase method as shown but without the aptamer present.

Our group has been exploring a different approach to aptamer-nanoMIP hybrids using a technique where the aptamer is modified along its length through the addition of polymerizable groups *via* modification of the thymine nucleobase. This was first demonstrated using a cocaine aptamer with quartz crystal microgravimetry (QCM) as the measurement system,^29^ supported by a study showing that the modified base integrates into the polymer matrix.^30^ This allows for the direct integration of the aptamer into the polymer matrix and hence the sequence acts as a true macro-monomer (Fig. 1-inset). This technique has proven effective for imprinting small molecules and protein targets alike.^31,32^ In all cases, the hybrid (aptaMIP) outperforms (in terms of affinity and specificity); both the corresponding non-aptamer bearing imprinted nanoparticles (nanoMIP), and the aptamer itself, often by orders of magnitude. Throughout these studies, incorporation of the aptamer into the polymer has been verified by spectroscopic techniques with comparable amounts of uptake across multiple studies, independent of the number and position of linkers.^29^

The validity of the technique in improving recognition is probably best demonstrated using a SARS-COV-2 spike protein targeting aptamer in the system. Here, the hybrid (*K*_D_ = 1.61 nM) outperformed both the MIP (24.4 nM) and aptamer (10.4 nM) against the wildtype while improving the selectivity against variants. Of interest the aptaMIP's affinity was below that of the ACE-2-Spike protein interaction suggesting potential for therapeutics.^33^

These aptaMIPs therefore highlight a “best-of-both-worlds” approach, where the aptamer, protected by a polymeric scaffold is protected from thermal, chemical and biological degradation,^29^ while the MIP receives a receptor unit with built-in and pre-determined binding properties towards the target of interest, as discussed above.

It can be hypothesised that the increase in aptamer performance within the MIP is due to the strand being entrapped within the polymer matrix, having formed its optimal binding conformation with the template target. This reduces any entropic effects (flexing of the sequence) and therefore increases affinity. Previous work in the area has supported this idea in that when the sequence was modified to have polymerizable units throughout the sequence, the performance was high. However, when the sequence was connected at only one end to the matrix (through a 5′-acrydite or a modified base at the 3′ end), all performance was lost.^29^ This prior work was performed using QCM analysis, a technique that is not as sensitive as others, in particular surface plasmon resonance (SPR), which has become a mainstay of MIP studies.^34,35^

Therefore, with these aptaMIPs, there is a fundamental question to be asked about how we link the nucleic acid sequence to the polymer to achieve best performance. This is vital as complexity of material design is a key factor in commercialisation opportunities, but also important experimentally as we move forward in developing this class of hybrid. Our prior work alludes to this but a full study exploring how the importance of both the position and number of linkers within an aptamer sequence, with respect to the performance (affinity and specificity) of an aptaMIP is required to appreciate the benefits of these hybrids.

The presented work highlights the value of correct positional selection of the attachment points and hindrance of sequence flexibility. We highlight how, through effective selection of linker placement, the recognition properties of an aptamer can be improved while utilising the polymer scaffold as a support that increases sequence stability to environmental conditions.

The nominal chosen target for this work was lysozyme, a small (14 kDa) enzyme that has been linked to multiple clinically relevant prognostic and therapeutic applications.^36^ It is found in multiple fluids including tears, bile, urine, serum, milk and even cerebral spinal fluid, at varying concentrations. It is also susceptible to change in many disease states and as such is a biomarker in a ranges of systemic diseases including HIV infection, tuberculosis and leukemia;^37^ as well as localised (*e.g.* conjunctivitis and dry eye disease in tears).^38^ In these biofluids, a range of enzymes exist which can risk degradation of biorecognition elements such as antibodies and aptamers, hence MIPs are a valid alternative.

While the choice of template for this materials study is essentially arbitrary (as in we are not focussing on development of a sensor, but on the structure–activity relationship of the polymer–nucleic acid hybrid), the key reason using lysozyme as a model template is that its DNA aptamer, which has not been previously used for any other aptaMIP process, was particularly appropriate due to its ideal sequence length (∼40 nucleotides in length). This favours rapid synthesis using a simple method and it contains multiple thymine residues along its length,^39^ enabling use to modify in regions (Fig. 1). These two key factors have allowed for a systematic examination of binding performance as a function of the number and location of polymerisation points.

## 2. Results and discussion

### 2.1. Synthesis

A previously published lysozyme aptamer sequence was selected for use in this study,^40^ which itself had been truncated from a slightly larger published structure.^38–40^ It was selected for several reasons. First, its length (42 bases) is like that of previously used AptaMIP sequences, allowing for a direct comparison.^31–33^ Secondly, it has suitably spaced thymine residues throughout, including bases near to each end, (Fig. 1) offering a range of modification positions. Finally, its binding affinity towards lysozyme is of a magnitude (*K*_D_ = 3.1 × 10^−8^ M) comparable to that of previously studied sequences for other protein targets.^32^

Out of the ten thymine residues within the 42-mer aptamer sequence, up to six with an even spacing from one another were selected as modification sites to allow for an effective comparison across different oligomers. Fig. 1 lists the eight oligomers made, along with the number and position of these modifications, which in each case comprised the commercially available carboxy-dT monomer, as reported previously.^29,32^ We selected T to demonstrated this, as the modified bases are easy to synthesise, when compared to the others. Modifying the phosphonamidite with a polymerizable group has proven exceptionally tricky to synthesise on A and C with yields between 3–5%. G is out of reach as the iodo-starting material is not available. Simply put, T is easy to work with and gave us the capabilities we needed. Also, our prior work was demonstrated using a polymerizable T as the linker attachment, so to maintain consistency and to limit variables (*i.e.* use of a different polymerizable base) into the system, we kept the chemistry the same, using carboxy-dT to replace the T's in the sequences. We also consider this as a suitable linker system as the attachment is away from the “working face” of the base so are less likely to affect aptamer–template interactions. We have not seen, in previous studies, any negative effects of the modifications affecting affinity.^29,33^

As described previously during the ammonia deprotection step following automated synthesis, the ester functionality on the modified thymine can convert to an amine^32^ (ESI,† Fig. S1). Due to the number of modifications used here, not all these residues in all the sequences were converted, as revealed by mass spectrometry (ESI,† Fig. S2). However, this was considered not to affect the overall MIP incorporation process as both functionalities are polymerizable. The aptamer strands listed in Fig. 1 were used to synthesise a range of aptamer-bearing molecularly imprinted nanoparticles (aptaMIPs), using the method developed previously by our group,^31^ the key principle of which is shown in Fig. 1 inset.

A control system (nanoMIP) was also synthesised under the same conditions. This was an imprint of lysozyme but in the absence of any DNA strand, so any recognition is down to the polymer matrix only, and not attributed to the presence of the aptamer. Here the polymer “recipe” is based on prior work by Piletsky, Canfarotta^41–43^ and ourselves^44^ using a mixture of basic, acidic and neutral monomers, that has shown itself capable of forming high-quality MIPs. This acts as a standard against which we can compare the performance effect of oligo incorporation. Prior work has shown that a control polymer bearing a non-polymerisable DNA strand (one that was not fixed into the polymer) produced poor recognition materials, so this control was not made.^29,45^

The polymerisation reactions proceeded as expected and produced a series of nanomaterials to which we assigned the codes displayed in Fig. 1. The polymerisation yields, which were obtained from a direct mass measurement from a dried-out sample, were comparable to previous work and ranged from 87–317 μg mL^−1^ (ESI,† Table S1). The yield variation can be explained by differences in solution volume used to wash the particles. They were found to be well within the requirements of this work as a total mass of only 300 μg was required in each case to prepare the SPR slides, with each dried sample resuspended in 1 mL of RO water prior to use.

Dynamic light scattering (DLS) was used to characterise the size of the nanoparticles. The resulting data are summarised in Fig. S3a, b and Table S2 (ESI†), with the distribution curves shown in the ESI.† An interesting observation is that the average nanoMIP size (98.94 ± 7.4 nm) is smaller than those of the eight AptaMIP polymers. This is a pattern observed previously,^33^ and suggests that the presence of the nucleic acid sequence results in an expanded nucleation site, which in turn increases the size of the polymer scaffold. However, the data indicates only a statistical difference in size for those strands with a single attachment point (AptaMIPs 6, 7 and 8). This can be potentially explained by those strands with two or more attachment points being more restricted, which possibly reduces the cavity size through providing a more hindered nucleation site and hence reducing the overall size of each nanoparticle.

### 2.2. Comparative binding performance

The binding performance of the MIPs toward target proteins was explored using surface plasmon resonance (SPR) spectroscopy. This technique is recognised as the gold-standard method for exploring host–guest binding interactions. Having been popular in antibody binding studies for many years, recently it has also been shown to be ideal for exploring MIP-template rebinding.^34,35^ While it is possible to affix the target template to the sensor surface and flow MIP nanoparticles over it, the preferred method, which we have done here, is to affix the nano-imprinted materials to the surface and exposing it to the target analyte. This reduces the effects of size heterogeneity in the attaching entity, and our analyte has a known mass which allows for the calculations of binding affinity to be calculated easier, without relying on estimations of particle size and mass.

Previous work in our group^31,32^ has demonstrated the benefits of using a carboxymethyl dextran-coated surface, to which nanoparticles can be attached *via* carbodiimide coupling (EDC/NHS), resulting in an even coating of MIPs. This procedure was followed again to generate the surface-attached nanoparticles. SPR binding studies in aqueous buffer at physiological pH were then carried out for each MIP in turn against the aptamer target protein lysozyme and two other proteins trypsin and BSA, which were used as negative controls. The equilibrium dissociation constant (*K*_D_) values derived from the SPR data are summarised in Table 1, with some of the sensorgrams discussed in turn in the following figures. The relevant *K*_a_ and *K*_d_ values obtained for the rebinding studies from which *K*_D_ are calculated at in Table S3 (ESI†).

**Table 1:** Equilibrium dissociation constants (*K*_D_) for the nanoMIP and AptaMIP-1 for binding the target template (lysozyme) and two other non-specific targets (BSA and trypsin) highlighting selectivity. Data extrapolated from SPR curves as shown representatively in Fig. 2 and Fig. S4, S5 (ESI). Brackets equals SD of *n* = 3

Polymer	*K*_D_ value (M)		
	Lysozyme	Trypsin	BSA
*NanoMIP*	*9.44 × 10* ^*−8*^ *(± 0.44 × 10* ^*−9*^ *)*	*6.42 × 10* ^*−6*^ *(± 0.56 × 10* ^*−6*^ *)*	*1.81 × 10* ^*−6*^ *(± 0.56 × 10* ^*−6*^ *)*
AptaMIP-1	1.28 × 10^−9^ (± 0.40 × 10^−9^)	4.68 × 10^−6^ (± 0.71 × 10^−6^)	9.00 × 10^−6^ (± 0.23 × 10^−6^)
AptaMIP-2	3.96 × 10^−9^ (± 0.79 × 10^−9^)	2.33 × 10^−6^ (± 0.34 × 10^−6^)	2.02 × 10^−6^ (± 0.25 × 10^−6^)
AptaMIP-3	1.20 × 10^−9^ (± 0.81 × 10^−9^)	9.75 × 10^−6^ (± 0.45 × 10^−6^)	2.06 × 10^−6^ (± 0.04 × 10^−6^)
AptaMIP-4	4.27 × 10^−8^ (± 0.87 × 10^−8^)	9.87 × 10^−6^ (± 0.36 × 10^−6^)	1.33 × 10^−6^ (± 0.56 × 10^−6^)
AptaMIP-5	8.34 × 10^−7^ (± 0.25 × 10^−7^)	8.95 × 10^−6^ (± 0.86 × 10^−6^)	1.17 × 10^−6^ (± 0.69 × 10^−6^)
AptaMIP-6	1.03 × 10^−6^ (± 0.66 × 10^−6^)	4.68 × 10^−6^ (± 0.96 × 10^−6^)	2.34 × 10^−6^ (± 0.86 × 10^−6^)
AptaMIP-7	1.53 × 10^−6^ (± 0.56 × 10^−6^)	4.89 × 10^−6^ (± 0.62 × 10^−6^)	3.00 × 10^−6^ (± 0.25 × 10^−6^)
AptaMIP-8	1.31 × 10^−6^ (± 0.06 × 10^−6^)	4.17 × 10^−6^ (± 0.72 × 10^−6^)	4.23 × 10^−6^ (± 0.53 × 10^−6^)

It should be noted that a non-imprinted polymer (NIP) has not been produced in this study. While this is a commonly used benchmark for traditional materials (bulk polymers, hydrogels films *etc.*), these cannot be produced using the solid-phase technique are utilised here. A silica bead with no attached template would not produce comparative polymers as there would be nothing for the nanoparticle to form around. Any material that did form would not have high enough affinity and be lost at the low temperature wash, leaving nothing to be collected at the higher temperature. It has also been noted that MIP and NIP have been repeatedly shown to have differing physical characteristics making a NIP an inferior comparator.^46^

This study focuses on the changes in affinity/selectivity observed when differing modified aptamer sequences are introduced so we have compared these against each other. The base control is a polymer, as highlighted above, lacks any aptamer addition.

**Fig. 2:**
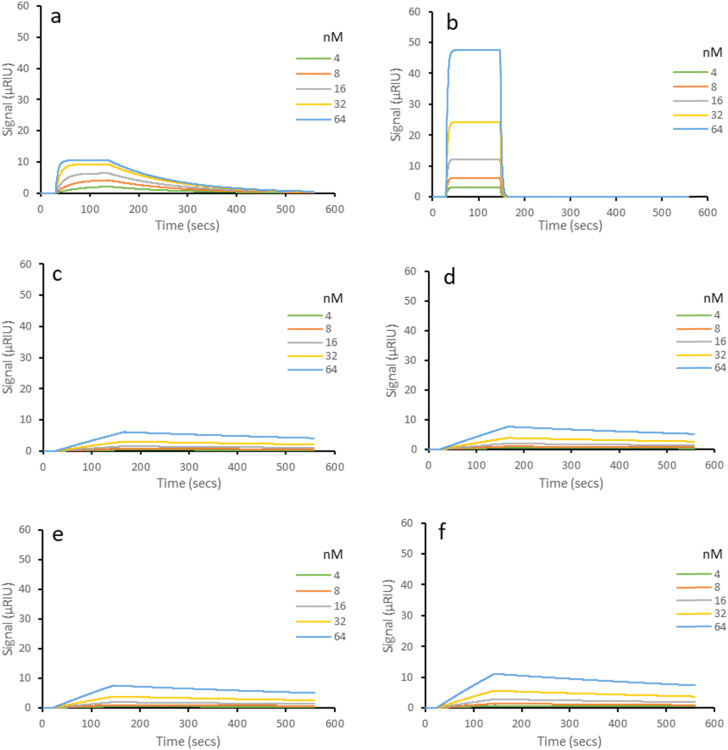
Representative SPR sensorgrams of molecular interactions of imprinted nanoparticles immobilized on carboxymethyl dextran hydrogel coated Au chips. Left column: NanoMIP. Right column: AptaMIP-1. The SPR running buffer (PBST) was a phosphate buffered saline made at 10 mM, pH 7.4, supplemented with 0.01% (v/v) Tween 20. Tween 20 is included to reduce non-specific binding during rebinding studies. Regeneration buffer was 10 mM glycine–HCl at pH 2. All rebinding at 25 °C. All experiments in triplicate. Range of five concentrations (between 4–64 nM) for each protein. (a) NanoMIP – lysozyme. (b) AptaMIP-1 – lysozyme. (c) NanoMIP – trypsin. (d) AptaMIP-1 – trypsin. (e) NanoMIP – BSA. (f) AptaMIP-1 – BSA.

Binding curves for NanoMIP and AptaMIP-1 are presented in Fig. 2. As expected, both NanoMIP and AptaMIP-1 bind lysozyme preferentially. The shapes of the rebinding curves towards the target molecule show a steep association when compared to the non-specific proteins, which supports a selective and high affinity process. The sharp dissociation curve for AptaMIP-1 (Fig. 2(b)) also suggests a homogeneity in the binding pocket recognition. This observation is consistent with the embedded aptamer “macromonomer” with its six attachment points adding greater selectivity to the binding pocket compared to NanoMIP, which relies on random complex formation within its sites and has a less pronounced tail-off in the dissociation process. As expected, the curves with trypsin and BSA are similar for nanoMIP and AptaMIP, with a low affinity association and a longer dissociation tail (less sharp dissociation) in both cases attributed to non-specific interactions.

The stark differences in the curves are reflected in the binding constants (Table 1), with AptaMIP-1 giving a ∼75-fold greater affinity towards lysozyme when compared to the NanoMIP. Although both materials exhibit selectivity for the imprinted target, the exceptional affinity of AptaMIP-1 results in high selectivity factors (*K*_D non-specific_/*K*_D specific_) of ∼3600 and ∼7000 observed for trypsin and BSA respectively, compared to ∼68 and ∼19 for the non-aptamer bearing nanoMIP.

The unfunctionalised lysozyme aptamer used in this study has a published *K*_D_ value of 3.1 × 10^−8^ M.^47,48^ and this is the baseline to which we are comparing. Interestingly, AptaMIP-1 gives a ∼25-fold increase in affinity for the same target. As implicated above, this can be likely explained by its six polymer attachment points locking the aptamer into an optimal pre-organised binding conformation, which reduces entropic effects.

This data supports our initial hypothesis that the use of an aptamer as a predetermined “monomer” improves the quality of the MIP system, while the “fixing” of the aptamer in a polymeric matrix to stabilise it improves the aptamers affinity as well. This matches prior work as discussed in the introduction (imprinting trypsin and moxifloxacin, where an aptamer was similarly modified throughout its sequence.^32,33^) and highlights the validity of the aptaMIP process creating hybrid materials as effective recognition systems.

This now leads to the question of whether the number, and location of, the linkers within the sequence play any part in the recognition performance. This question can be answered by analysing the data for the other MIPs made in this study as discussed below.

Fig. S4a (ESI†) shows the binding curves attributed to AptaMIPs 2 through 5 towards lysozyme. The results for the non-specific targets (BSA and trypsin) can be found in the ESI† (Fig. S4b). Those for AptaMIP-2 and AptaMIP-3 (Fig. S4a-a and b-b, ESI† respectively) exhibit the same defined association/dissociation profiles to those observed with AptaMIP-1, suggesting similar performance profiles, though some slight tailing for AptaMIP-2 is observed. This is borne out by the binding data, with both AptaMIP-2 and AptaMIP-3 giving *K*_D_ values in the low nM range (Table 1), that are comparable to AptaMIP-1.

This suggests that the aptamer sequence within both AptaMIP-2 (four linkers positioned evenly throughout) and AptaMIP-3 (three linkers evenly spaced at either end and centre) can also be suitably fixed and pre-organised for strong binding. In fact, AptaMIP-3 exhibits the lowest *K*_D_ value in this study (1.20 × 10^−9^ M, albeit within the SD of AptaMIP-1). The cross-reactivity studies of these two materials matches that seen by AptaMIP-1, in that exceptional selectivity is observed (selectivity factors between 500–8000 in cross-reactivity studies – Table 1). The profiles of binding to the alternative analytes are as expected with slow association/dissociation observed (Fig. S4b-e, f and b-i, j, ESI†).

These data combined with those of AptaMIP-1 supports the idea that the aptamer needs to be fixed throughout its length to be effectively held into a good binding conformation. This is confirmed by the data from AptaMIPs 4 through 8.

Fig. S4a–c (ESI†) shows the rebinding profile of AptaMIP-4, which has three linkers close to the centre of the aptamer sequence (Fig. 1). This leaves some of the sequence “loose” at either end (nine bases at 3′ and fourteen bases at 5′) and able to flex. While the binding curves exhibit excellent profiles, the intensity of the signal is vastly reduced, suggesting the binding performance (amount of analyte bound) is affected as a result. This is reflected in the resultant *K*_D_ value of 4.27 × 10^−8^ M, which is a significant drop (33- and 35-fold) on the best performing AptaMIPs (1 and 3) respectively, but still two-fold better than the nanoMIP (non-aptamer bearing). This slightly lower affinity can be explained by the “macromonomer” sequence being able to move within the pocket, and not locked into place. This supports the hypothesis of “fixing” the aptamer to reduce entropic effects. The non-specific binding of alternate analyte protein (Fig. S4b-g and b-k, ESI†) values are comparable to all other synthesised polymers (∼mid 10^−6^ M) suggesting the same non-specific mechanism as all other polymers. Interestingly, AptaMIP-4 approximates the affinity of the aptamer on its own. Interestingly where we have previously fixed an aptamer in a manner observed in AptaMIP 4 (with part of the sequence free to move at either end) as seen with trypsin,^32^ the observed performance increase is of similar order of magnitude and intensity – supporting the idea of the “loose” ends being able to move.

Fig. S4a–d (ESI†) shows the data for AptaMIP-5 which has the sequence affixed within the polymer through linkers at each end only (position 1 and 6 – Fig. 1) to allow significant flexibility of the sequence within the cavity. The cross-reactivity is, as expected, non-specific in nature (Fig. S4b-h and b-l, ESI†) as observed across all similar materials. However, the calculated *K*_D_ value towards lysozyme is an order of magnitude lower than that for AptaMIP-4 (Table 1) which in contrast allows for flexibility at each end of the sequence while fixing the middle; and is two orders of magnitude worse (∼650-fold) than that for AptaMIP-1 which fixes throughout and AptaMIP-3 which fixes in the two same positions but a third central one. The observed binding profiles (Fig. S4a–d, ESI†) show reasonably good association, though overall the degree of interaction is reduced suggesting limited analyte uptake, with a significant tail in dissociation suggesting heterogeneity within the pocket. Below the 64 nM curve, the association curves are less sharp, suggesting a heterogeneity in any formed cavity affinities. This further supports the hypothesis that allowing the aptamer macromonomer (or part thereof) freedom to move within the binding cavity post synthesis reduces overall affinity and performance. It also suggests that “locking” the central part of the sequence is important as AptaMIP-2 (which foregoes a central linker leaving a flexible 14 base section shows slight tailing in dissociation (Fig. S4a-a, ESI†)), unlike those that are affixed at the central section (AptaMIP-1 and AptaMIP-3).

The three MIPs with just one sequence connection point, at the 3′ end (AptaMIP-6), 5′ end (AptaMIP-7) and centrally (AptaMIP-8) continue the same trend of poorer binding properties with fewer effective recognition sites. The binding curves for these polymers and lysozyme are shown in Fig. S5a (ESI†), with the those for the control proteins BSA and trypsin shown in Fig. S5b in the ESI.† The profile for the 3′-linked sequence (Fig. S5a-a, ESI†) suggests heterogeneity in the binding cavity. The overall magnitude of signal is also low, which suggests poor uptake of analyte, while the association rate is clearly slower, as is the dissociation rate. The same pattern is observed in the sequence fixed in the centre position (Fig. S5a–c, ESI†), although a slightly higher association rate is seen.

Interestingly, the binding profile of the 5′-linked sequence (Fig. S5a and b, ESI†) suggests a better recognition profile than the other two single-point linked polymers, though poor analyte uptake is observed and at lower concentrations association rate slows down. In contrast to what is found for the other MIPs (Fig. 2 and Fig. S4, ESI†) the binding profiles for the two non-specific analytes (Fig. S5bd–i, ESI†) are similar to those for lysozyme, suggesting that non-specific binding is playing a larger part of the overall acquired binding signal. Once again, these SPR results are borne out by the *K*_D_ values for these three MIPs, with all three being poorly selective over the non-template proteins (Table 1) and having low binding affinities (*K*_D_ values are ∼1000-fold higher compared to the fully fixed aptamer – AptaMIP-1). Selectivity factors (*K*_D non-specific_/*K*_D specific_) are exceptionally poor (*i.e.* AptaMIP-6 exhibits and SF of ∼2.3 for BSA and 4.54 for trypsin) compared to ∼7000 (BSA) and ∼3600 (trypsin) for AptaMIP-1. Selectivity factor is a key indicator of good imprinting but also in this case how well the aptamer within the binding pocket can act as a macromonomer.

Some interesting comparisons can be made which support the hypothesis of the aptamer flexing within a cavity. The initial clear observation is that materials with embedded aptamers held by only one linker perform poorly. This is interesting as the principle of alternative aptamer MIP hybrid systems (as discussed in the introduction) are built around a single linker point with a polymer matrix built around it.^26–28^ These studies do show improvements in binding over component parts, as we do here, but nearly all rely on electrochemical detection which gives limited data on the nature of association/dissociation, so it is difficult to comment on the nature of the interactions in these other studies.

Of interest, AptaMIP-5 (bound at each end) gives a *K*_D_ of 8.34 × 10^−7^ M, which is only a slight improvement on the performance on those bound at either end only (1.03 × 10^−6^ M and 1.53 × 10^−6^ M for AptaMIP-6 and AptaMIP-7 respectively). This is surprising as it is easy to picture the aptamer folding in upon itself if only linked in one place but flexing when held in two places is not immediately easy to visualise how much it can or will move. This is further supported by data that shows that fixing the centre part of the aptamer sequence is critical to performance as highlighted by AptaMIP-3 which through the addition of a centre point linker improves the affinity for the target protein 700-fold over the AptaMIP-5. Further evidence of the importance of multiple linkers is seen comparing AptaMIP-8 (single centre point) *vs.* AptaMIP-4 which also is centre linking, but with three points. Using the latter gives a 30-fold improvement in *K*_D_ over a single centre point, by holding more of the centre of the sequence in place.

**Fig. 3:**
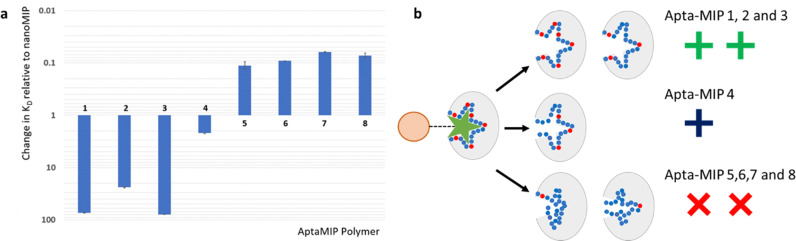
(a) Relative changes in *K*_D_ respective to the baseline nanoMIP control (Table 1) for each aptaMIP binding to its target ligand lysozyme. A decrease on the *Y*-axis indicates an improvement in binding affinity. An increase signifies poorer performance. *Y*-Axis presented in log scale for clarity. *N* = 3. Actual *K*_D_'s for each aptaMIP presented in Table 1. (b) Proposed effect of positional linkers on AptaMIP binding performance. After the imprinting process, aptamers that are fixed throughout the sequence perform well as the oligo is held in a set position. (Top). Those that offer some oligo flexibilities are still able to recognise the analyte but with limited affinity (middle), while those which use limited attachments offer negative effects (bottom) when compared to the control polymer nanoMIP and aptamer alone. The is suggested to due to the aptamer, which acts as the recognition element within the formed binding pocket losing stability away from its binding conformation and the oligo collapses.

Fig. 3 summarises the data presented in Table 1 for the rebinding to each AptaMIP of the target ligand lysozyme presented relative to the control NanoMIP which bears no aptamer sequence. This also allows for visualisation of each polymer relative to each other. There is a clear pattern linking the number and position of the polymerizable linker groups to the performance of the polymers. The data clearly shows that fixing the sequence throughout the whole length is preferable (AptaMIP-1 and AptaMIP-3), while allowing any flexibility in the sequence, be it at either end, or in the centre reduces affinity significantly from the fully fixed sequence, and negatively affects selective rebinding (compared to the control nanoMIP).

From prior studies we know that incorporation of the nucleic acid sequences is consistent in terms of sequence becoming entrapped in the polymer, so variation between sequence uptake is not likely to impair upon the analytical data obtained, especially considered the orders of magnitude differences. We also can assume that if the sequence wasn’t bound then performance would match the control. The solid-phase method doesn’t allow for nanoparticles to be made that aren’t imprinted (the wash step removes low-affinity and unbound materials) so we can safely assume that the differences observed are due to changes in the binding pocket structure caused by the sequence addition.

This lysozyme aptamer requires clear fixing of the centre of the sequence. Any non-bound sections in the centre of the sequence seems to affect recognition as seen in AptaMIP-2 (lesser extent) and AptaMIP-5 (greater extent). Interestingly, the data suggests that using three linkers (each end and a centre) is enough to lock the sequence in place. This has implications for the technology moving forward as it reduces the amount of modified linker that is needed which in turn will reduce cost. This may vary for different aptamers, but this work is a good starting point as an indicator of what may be required.

### 2.3. Application in sensitive template detection

**Fig. 4:**
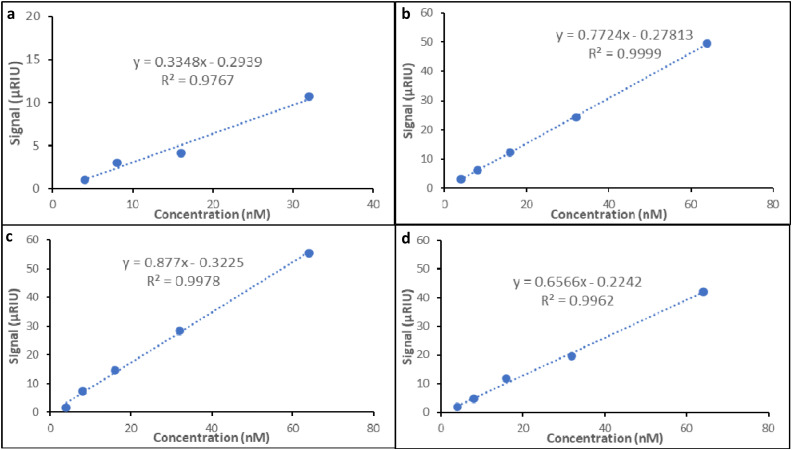
Elucidation of theoretical limit of detection for SPR sensor using representative signal. Relative signal *versus* concentration. (a) Lysozyme binding to nanoMIP, (b) Lysozyme binding to AptaMIP 1, (c) Lysozyme binding to AptaMIP 2, (d) Lysozyme binding to AptaMIP 3. Note: only four points were used for the nanoMIP LOD calibration, as this was the linear range of the data. Note: SD's are not shown based on how the data was collated – refer to Table 1 as indicator of variance in workstream.

Based on the structural work here, we have applied the control material (nanoMIP) and the best performing materials (aptaMIPs 1, 2 and 3) as recognition elements in an SPR-based sensor. While this is not the core drive of the manuscript it is important to validate the application of this materials and highlight the importance of a high-quality recognition element. This data is an extension of the SPR responses from in Fig. 2(a), (b) and Fig. S4a-a, a-b (ESI†) (for the nanoMIP, aptaMIP 1, aptaMIP 2 and aptaMIP 3, respectively) and presented in Fig. 4, with a linear range into the mid nM and above – well within the useful biological range.^36–38^

Using these materials as recognition elements, we calculated the theoretical LOD for lysozyme for the SPR-based sensor of 0.88 nM, 0.36 nM, 0.37 nM, and 0.34 nM, (Table 2), for the nanoMIP, aptaMIP 1, aptaMIP 2 and aptaMIP 3, respectively. Clearly, the better performing polymers (aptaMIP 1, 2 and 3) provide linear response and superior LODs. It should also be noted that only four points were used in the calculation of the theoretical LOD for the nanoMIP (Fig. 4(a)), this is due the saturation of target at higher concentration on the sensor and can be seen in Fig. S5 (ESI†). This highlights a further benefit of aptamer incorporation – the materials offer more even binding over a concentration range suggesting more defined recognition.

**Table 2:** Theoretical limit of detection (nM) of an SPR sensor that incorporate either the nanoMIP and aptaMIP (1, 2, and 3) materials and LODnanoMIP/LOD_aptaMIP_ calculated ratios

Polymer	Theoretical LOD (nM)	Ratio LOD_nanoMIP_/LOD_aptaMIP_
NanoMIP	0.88	
AptaMIP-1	0.36	2.43
AptaMIP-2	0.37	2.57
AptaMIP-3	0.34	2.57

The three aptaMIPs that showed the best affinity (aptaMIP 1, 2, 3), showed the lowest LOD thus the lowest sensitivity. These estimates show that the identical sensor surfaces coated with the high-performing aptaMIP (1, 2 and 3) particles can detect lower concentrations of the target molecules, compared with the nanoMIP. The ratio of the LOD_nanoMIP_/LOD_aptaMIP_, is 2.43, 2.57, 2.57 for aptaMIP 1, 2, and 3, respectively (Table 2). This shows that not only are the performance of the three aptaMIPs is approximately the same, but that the is an approximately a 2.5-fold sensitivity improvement of the LOD detection against the nanoMIP, validating the importance of recognition element selection and highlighting why improving the affinity of a MIP is critical in application.

This highlights that the aptaMIP particles demonstrate superiority as a reusable synthetic recognition material and that the improvement performance, by approximately 2.5-times is significant enough in analytical terms to be the difference between a positive and negative results. It should be pointed out the theoretical LODs for the remaining aptaMIPs (4, 5, 6, 7, and 8) could not be detected using the same methodology, this is due to limited discrimination between the poorer quality materials and non-specific binding on the control channel; alongside saturation caused by the poor selective rebinding of the target molecule within these SPR-based sensor systems and can be seen in Fig. S6 (ESI†). This serves to additionally highlight the importance of developing high-quality recognition materials, as well as the importance of our design methodology, ensuring that the modified thymine-base is present in the right locations within the aptaMIP hybrid system.

## 3. Conclusion

The data clearly shows the benefits of aptamer incorporation into the MIP. Performance of the best aptaMIP when compared to the nanoMIP (without an oligo incorporated) is improved by ∼75 fold Likewise, the hybrid material outperforms the aptamer on its own. This matches prior work and observations that these hybrids, the marriage of technologies, improves affinity over both components (MIP and aptamer only).

Focussing on the comparators in number and location of linkers, or how the aptamer is fixed with the MIP, the data presented here offers a hypothesis that, if not preorganised and locked in place, an aptamer within a MIP is able to flex/move/collapse within the imprinted cavity, which is detrimental to binding performance. This is shown schematically in Fig. 3(B), which demonstrates the effect of having different numbers and locations of polymer attachment points.

We theorise that without effective linking throughout the sequence, the oligomer will flex and move after template removal. In fact, as the polymer scaffold will bear its own electrostatic charge arising from the chemical functionality (acrylic acid/acrylamides) of the monomers, this could have a significant effect on the shape of the aptamer when it is not fully attached, forcing it into non-favourable binding conformations. This might be the reason why the affinity level can be reduced to that of non-specificity, *i.e.* lower than that found for the MIP or aptamer alone. Even if the sequence can return to an effective binding conformation in the presence of the target, its lack of preorganisation would prevent strong association and its resting conformation may even partially block the cavity, making the binding affinity less than that for the nanoMIP, which does not contain any aptameric sequence. We plan to further explore this by altering the composition of the polymer, and potentially through FRET analysis, but these studies are outside the scope of the currently presented work.

This could also be why, in the electrochemical surface bound sensor studies discussed in the introduction above,^26–28^ the significant differences shown here are not observed as prominently, given the nature of the polymer matrices used. It would be interesting to see if it was possible to combine both techniques and use an aptamer modified in such a way that it could be electropolymerized into the surrounding scaffold – this may improve performance further.

What is also clear is that the number of attachment points and their position in the sequence is vital to the binding performance of an AptaMIP, and by association how effective this method is at improving aptamer recognition. In this work, a modified thymine was used to generate the linkers as the aptamer has thymine residues throughout its sequence. However, in other aptamers, modifications to other nucleobases may be required (*e.g.* where a thymine may not be available at the end of a sequence or at specific points throughout). Therefore, priority needs to be given to modification of other bases, allowing for selective modifications wherever they are needed. Our groups are currently exploring these ideas. Also, for much longer aptamers that adopt various secondary structures, positional selection for placing linkers will be key to ensure they do not interfere with formation of the required structures.

In terms of sensing, these findings offer a way to provide highly selective recognition elements, while the design of these materials, through further polymer optimisation, can be considered in a range of optical, gravimetric, and electrochemical platforms.

The process of stabilising an aptamer within an imprinted cavity offers significant benefits in terms of affinity improvements over the components used individually (*i.e. versus* nanoMIP and aptamer alone) and increasing the stability of the bound nucleic acid in environments where degradation may be observed (low/high pH, high temperature and the presence of enzymes). Prior studies have shown this.^29,30^ Therefore aptaMIP technology has significant potential for future healthcare and environmental applications, exploiting the benefits of the “best-of-both-worlds” approach that this technique brings, not only in simple diagnostics (as demonstrated here) but also for improving therapeutics and drug delivery, where the use of a polymer scaffold could increase load capacity and improve agent stability.

## 4. Experimental section

### 4.1. Materials

All chemicals and solvents were of analytical or high-performance liquid chromatography (HPLC) grade and were used without further purification. Solvents used in materials synthesis and analysis (acetone, acetonitrile (dry), methanol) alongside additives and buffers (dipotassium phosphate, disodium phosphate, ethanolamine, ethylenediaminetetraacetic acid (EDTA), potassium chloride, sodium dodecyl sulphate (SDS), sodium hydroxide and Tween 20) were purchased from Fisher Scientific UK Ltd, Loughborough, UK. Water was provided *via* Milli-Q system (Merck, Dorset, UK).

Monomers (acrylic acid (AAc), *N*,*N*′-methylenebisacrylamide (BIS), *N*-hydroxysuccinimide (NHS), *N*-isopropylacrylamide (NIPAm), *N-tert*-butylacrylamide (TBAm)); and reaction agents (1-ethyl-3-(3-dimethylaminopropyl)carbodiimide (EDC), 3-aminopropyltrimethyloxy-silane (APTMS), ammonium persulfate (APS), glutaraldehyde (GA), glycine and tetramethylethyldiamide (TEMED)), were all purchased and used without purification from Merck, Poole, Dorset, UK. Protein targets (bovine serum albumin (BSA)), lysozyme, and trypsin were purchased from Fisher Scientific UK Ltd, Loughborough, UK. Glass beads were purchased from Supelco, Bellafonte, PA, USA. Carboxy-dT-CE phosphonamidite were purchased and used without purification from LGC Link, Bellshill, Lanarkshire, UK. Double-distilled water (DD-H_2_O) was produced on site.

### 4.2. Synthesis of polymerizable aptamer sequence

The lysozyme aptamer sequence was chosen from the literature,^40,47,49^ with the shortened version used as it could offer the desired properties required for this study.^48^ The selected sequence was 5′-ATC AGG GCT AAA GAG TGC AGA GTT ACT TAG-3′.

Modified versions of the sequence were synthesized and purified using the procedure reported previously.^29,31,33^ The modifications involved the replacement of selected thymine bases with the commercially available carboxy-dT-CE phosphoramidite.^31–33^ The synthesized oligomers were deprotected and released from the support by treatment with concentrated aqueous ammonia at 60 °C for 24 h. The solutions were concentrated to dryness, resuspended in water, and purified by semi preparative HPLC on an Agilent 1260 infinity system with a Phenomenex Clarity 5 μm Oligo-RP LC 250 × 10 mm column. Collected fractions were desalted using NAP-10 columns (GE Healthcare) and oligo purity was determined by analytical HPLC on an Agilent 1260 infinity system with a Phenomenex Clarity 5 μm Oligo RP LC 250 × 4.6 mm column. Oligonucleotide masses were verified using a Waters Xevo G2-XS, and concentrations were determined by optical density at 260 nm using a BioSpec-nano micro-volume UV-Vis spectrophotometer (Nanodrop, Shimadzu), and the Beer–Lambert law, with extinction coefficients obtained from OligoAnalyzer (Integrated DNA Technologies).

### 4.3. Preparation of lysozyme-derivatized glass beads as affinity media

Glass beads (30 g, 75 μm diameter) were activated by boiling in 4 M NaOH (24 mL) for 15 minutes, then washed thoroughly (8 × 100 mL DD-H_2_O) to reduce the solution pH to 7. The beads were rinsed (2 × 100 mL acetone) before drying at 80 °C for three hours. Once dry, the beads were placed in a 12 mL solution of APTMS (3%, v/v) in anhydrous toluene in 12 hours at 60 °C (oven) to add an amine functionality. Following this incubation, the beads were washed (8 × 100 mL acetone + 2 × 100 mL methanol) and dried at 150 °C for an hour.

The beads were then added to a 7% (v/v) glutaraldehyde solution (15 mL) for 2 hours at room temperature (sealed in nitrogen), before 7.5 mg of lysozyme in 15 mL phosphate-buffered saline (PBS, 10 mM) pH 7.4, was added. This was left to incubate at room temperature overnight, maintaining the sealed nitrogen atmosphere. After this, the beads bearing the lysozyme template were washed thoroughly (8 × 100 mL DD-H_2_O) and dried at room temperature under vacuum. Once dry, the glass beads were immediately used for the polymer synthesis step, without any storage. Note: beads can be stored at 4 °C for up to four weeks but it is recommended to use them immediately.

### 4.4. Solid-phase supported synthesis of lysozyme imprinted nanoMIPs and hybrid-MIPs

A polymerisation mixture consisting of NIPAm (20 mg). BIS (1 mg), and AA (2.2 μL) in 49 mL of DD-H_2_O was made up, to which 250 μL of ethanol containing 17 mg of TBAm was added (the latter was made separately). This mixture was made up to a total volume to 50 mL, degassed under vacuum while sonicating for 10 minutes. Following this, the solution was bubbled with nitrogen gas for 20 minutes. This recipe was selected as it is known to be effective at creating imprinted nanomaterials^33,44^ and will enable us to generate an effective control baseline polymer (nanoMIP) against which we can compare any changes caused by the presence of the aptamer.

Concurrently 30 g of lysozyme-bearing glass beads (the solid phase) were transferred into a 100 mL sealable bottle and degassed by purging with N_2_ for 10 minutes. The prepared monomer solution was added, followed by 12.5 μL of TEMED and 15 mg of APS dissolved in 250 μL of DD-H_2_O to initiate the polymerisation reaction, which was left to run for one hour. Every ten minutes the solution was gently swirled. This was completed at room temperature (20 °C).

After the synthesis, the beads were filtered through a 11 μm filter paper and washed (8 × 30 mL DD-H_2_O at ambient temperature). Then the beads were heated in 40 mL of DD-H_2_O at 60 °C, then filtered again through a 11 μm filter paper. Three further washes of 20 mL DD-H_2_O at 60 °C were carried out, with all the solutions (∼100–120 mL bearing the eluted nanoMIPs but leaving the template bearing beads behind) collected. This solution was cooled and stored in at 4 °C until required.

To produce the hybrid AptaMIPs, the same protocol above was followed except that in the initial step, where the initial monomer solution consisted of a solution of 1.74 μmol of the aptamer in 10 mL of DD-H_2_O, to which a polymerisation mixture consisting of NIPAm (20 mg). BIS (1 mg), and AA (2.2 μL) in 39 mL of DD-H_2_O was added. To this solution, 250 μL of ethanol containing 17 mg of TBAm was added, then made up to a total volume to 50 mL, before degassing, sonicating and nitrogen bubbling.

In all systems the mixture was left to equilibrate for 15 minutes before reaction was started to allow for the template–monomer (template–aptamer) complexes to form.

### 4.5. Nanoparticle characterisation

Effective hydrodynamic diameters (dh) of the particles were determined using dynamic light scattering (DLS) with a NanoBrook Omni spectrometer (Brookhaven, United States) at 25 °C. Five measurements per sample were made with a 10 second time interval and triplicate samples. Particle sizes were determined using Particle Solutions (v2.6) software.

To calculate the concentration of the synthesised particles, 3 mL of the nanoparticle solution was evaporated to dryness at 60 °C. The mass of the dried particles was then measured and divided by three to reveal the concentration in μg mL^−1^. Five samples were analysed to obtain an overall average.

### 4.6. Immobilisation of the SPR sensor surface

Surface plasmon resonance (SPR) was utilised to evaluate the affinity and specificity of the imprinted nanoparticles towards lysozyme and two other proteins trypsin and bovine serum albumin (BSA) were used to test for cross-reactivity. A Reichert 2SPR system (Buffalo, NY, USA) was employed for this. Carboxymethyl dextran hydrogel coated Au chips were selected for immobilisation. A precondition step was used until a stable baseline was reached, using PBST (PBS pH 7.4 and 0.01% Tween 20) at 10 μL min^−1^. This flow rate of 10 μL was maintained throughout the immobilisation process. 40 mg EDC and 10 mg NHS was dissolved in 1 mL water, sonicated for 10 minutes, degassed briefly using vacuum and immediately injected onto the sensor chip surface for 6 minutes under flow. This was followed by 300 μg of the nanoparticles dissolved in 1 mL of the running buffer (PBST) and 10 mM sodium acetate (added in order to activate the NH functional groups within the MIP NP), allowing for and EDC–NHS coupling to occur.

The nanoparticle solution was injected only to the left channel (the left channel is the working channel, right channel is the reference) of the surface for one minute followed by an eight-minute flow of a quenching solution (1 M ethanolamine, pH 8.5) to deactivate carboxyl groups and to wash away the unbound nanoparticles. Once the immobilisation procedure had been completed, a continuous flow of running buffer (PBST) at 10 μL min^−1^ was maintained. Rebinding assays were carried out after a stable baseline was achieved.

### 4.7. Kinetic analysis using SPR

For each rebinding assay, the flow was set at 10 μL min^−1^. An injection of running buffer PBST (blank) was added for two minutes (20 μL) across the sensor, followed by the running buffer for five minutes. The binding kinetics of an individual aptaMIP/nanoMIP to the selected target protein (lysozyme) was determined from serial dilutions (five concentrations between 4–64 nM). Each dilution was injected for two minutes (association) followed by PBST running buffer for 5 min (dissociation), then a regeneration step using a regeneration buffer (10 mM glycine–HCl, pH 2) for one minute followed by PBST for one minute, then the steps were repeated for all dilutions.

The signal measured from each of five concentrations of the target protein was fitted to a 1 : 1 bio-interaction model (Langmuir fit model) utilizing TraceDrawer Software. Signals from left channel (working) were subtracted from signal from their respective reference channel (right channel). Association rate constant (*k*_a_), dissociation rate constant (*k*_d_), and maximum binding (*B*_max_) were fitted globally, whereas the BI signal was fitted locally. This enabled the equilibrium dissociation constant (*K*_D_) to be calculated from the ratio *k*_d_/*k*_a_. Specificity for the aptaMIP/nanoMIP particles was investigated by repeating the SPR kinetic analysis but using two non-target proteins (trypsin and BSA) at the same concentrations instead. All analyses were carried out at 25 °C.

## Author contributions

Sullivan: investigation, verification, writing – original draft preparation, methodology; Allabush: investigation, methodology; Mendes: funding acquisition, supervision; Tucker: supervision, writing – original draft preparation; Turner: conceptualisation, funding acquisition, project administration, writing – original draft preparation, methodology, supervision.

## Data availability

ESI† is available from the publishing website. The raw and processed data underlying this article will be shared on reasonable request to the corresponding author.

## Conflicts of interest

The authors have no conflicts of interest.

## Supplementary Material

TB-013-D4TB02475C-s001
