# Supplementary material for: Incorporation of polymerizable linkers into aptamers for high-affinity nanoscale molecularly imprinted polymer hybrids: analysis of positional selectivity
Source: J Mater Chem B. 2025 Mar 11;13(14):4374–85. doi: 10.1039/d4tb02475c (PMC11905881; doi:10.1039/d4tb02475c)
Supplement: TB-013-D4TB02475C-s001 [file TB-013-D4TB02475C-s001.pdf]

This journal is © The Royal Society of Chemistry 2025

Supplementary Information

Incorporation of Polymerizable Linkers into Aptamers for High-Affinity Nanoscale Molecularly Imprinted Polymer Hybrids: Analysis of Positional Selectivity

Mark V. Sullivan[a], Francia Allabush[b], Paula M. Mendes[b], James. H. R. Tucker[c] & Nicholas W. Turner[a]

a. Department of Chemistry, University of Sheffield, Sheffield, S3 7HF, UK.
b. School of Chemical Engineering, University of Birmingham, Edgbaston, Birmingham, B15 2TT, UK
c. School of Chemistry, University of Birmingham, Edgbaston, Birmingham, B15 2TT, UK

*E-mail: nicholas.turner@sheffield.ac.uk

Table of Contents:

Table S1: Synthesis Yields of polymeric nanoparticles

Table S2: DLS particle sizing for synthesised MIPs. N = 5.

Table S3: K_a and K_d values obtained for the rebinding studies from which K_D are calculated.

Figure S1: Amination of ester during synthesis

Figure S2: Example LC-MS spectra of sequence synthesis highlighting non-converted masses. Example given is AptaMIP-1.

Figure S3a: DLS particle sizing for synthesised MIPs. Representative Curves.

Figure S3b: DLS particle sizing for the synthesised MIPs. N = 5. Error bars highlight sample particle size distribution (Supplementary Figure S3). Truncated y-axis for clarity.

Figure S4a Representative SPR sensorgrams of molecular interactions of imprinted nanoparticles AptaMIP 2,3,4 and 5 immobilized on carboxymethyl dextran hydrogel coated Au chips towards Lysozyme

Figure S4b: Representative SPR sensorgrams of molecular interactions of imprinted nanoparticles AptaMIP 2,3,4 and 5 towards BSA and Trypsin.

Figure S5a Representative SPR sensorgrams of molecular interactions of imprinted nanoparticles AptaMIP 6,7, and 8 immobilized on carboxymethyl dextran hydrogel coated Au chips towards Lysozyme

Figure S5b: Representative SPR sensorgrams of molecular interactions of imprinted nanoparticles AptaMIP 6,7 and 8 towards BSA and Trypsin.

Figure S6. Response curves for poorer-quality MIP systems

Supplementary Information

Table S1: Synthesis Yields of polymeric nanoparticles

MIP	mass (µg mL^{-1})
nanoMIP	90 (± 3)
AptaMIP-1	143 (± 7)
AptaMIP-2	136 (± 6)
AptaMIP-3	87 (± 5)
AptaMIP-4	213 (± 11)
AptaMIP-5	217 (± 16)
AptaMIP-6	237 (± 15)
AptaMIP-7	317 (± 17)
AptaMIP-8	287 (± 19)

Table S2: DLS particle sizing for synthesised MIPs. N = 5.

MIP	Size (nm)
nanoMIP	98.94 (± 7.4)
AptaMIP-1	101.48 (± 0.8)
AptaMIP-2	106.13 (± 6.3)
AptaMIP-3	105.47 (± 5.6)
AptaMIP-4	102.80 (± 2.8)
AptaMIP-5	101.91 (± 1.9)
AptaMIP-6	114.98 (± 1.1)
AptaMIP-7	113.76 (± 1.0)
AptaMIP-8	119.55 (± 9.5)

Table S3: K_a and K_d values obtained for the rebinding studies from which K_D are calculated.

	Lysozyme	
**Polymer**	Ka	Kd
NanoMIP	8.75×10^{5} (± 0.87×10^{4})	8.26×10^{-2} (± 0.58×10^{-3})
AptaMIP-1	1.60×10^{7} (± 0.23×10^{6})	2.05×10^{-2} (± 0.33×10^{-3})
AptaMIP-2	1.35×10^{7} (± 0.34×10^{6})	5.35×10^{-2} (± 0.43×10^{-3})
AptaMIP-3	1.36×10^{6} (± 0.64×10^{5})	1.63×10^{-3} (± 0.90×10^{-4})
AptaMIP-4	4.97×10^{5} (± 0.57×10^{4})	2.12×10^{-3} (± 0.40×10^{-4})
AptaMIP-5	5.01×10^{5} (± 0.36×10^{4})	4.18×10^{-1} (± 0.89×10^{-2})
AptaMIP-6	2.55×10^{5} (± 0.41×10^{4})	2.62×10^{-1} (± 0.45×10^{-2})
AptaMIP-7	2.35×10^{5} (± 0.72×10^{4})	3.60×10^{-1} (± 0.33×10^{-2})
AptaMIP-8	2.07×10^{5} (± 0.34×10^{4})	2.71×10^{-1} (± 0.79×10^{-2})
	**Trypsin**	
**Polymer**	Ka	Kd
NanoMIP	4.47×10^{4} (± 0.75×10^{3})	2.87×10^{-1} (± 0.32×10^{-2})
AptaMIP-1	5.88×10^{4} (± 0.62×10^{3})	2.75×10^{-1} (± 0.10×10^{-2})
AptaMIP-2	8.81×10^{4} (± 0.27×10^{3})	2.05×10^{-1} (± 0.94×10^{-2})
AptaMIP-3	8.44×10^{4} (± 0.60×10^{3})	8.23×10^{-1} (± 0.96×10^{-2})
AptaMIP-4	8.54×10^{4} (± 0.39×10^{3})	8.43×10^{-1} (± 0.67×10^{-2})
AptaMIP-5	9.06×10^{4} (± 0.98×10^{3})	8.11×10^{-1} (± 0.70×10^{-2})
AptaMIP-6	7.22×10^{4} (± 0.92×10^{3})	3.38×10^{-1} (± 0.66×10^{-2})
AptaMIP-7	7.36×10^{4} (± 0.81×10^{3})	3.60×10^{-1} (± 0.23×10^{-2})
AptaMIP-8	7.81×10^{4} (± 0.40×10^{3})	3.26×10^{-1} (± 0.87×10^{-2})
	**BSA**	
**Polymer**	Ka	Kd
NanoMIP	2.68×10^{4} (± 0.58×10^{3})	4.86×10^{-1} (± 0.52×10^{-2})
AptaMIP-1	9.68×10^{4} (± 0.33×10^{3})	8.71×10^{-1} (± 0.76×10^{-2})
AptaMIP-2	1.68×10^{4} (± 0.10×10^{3})	4.86×10^{-1} (± 0.47×10^{-2})
AptaMIP-3	2.51×10^{4} (± 0.24×10^{3})	5.17×10^{-1} (± 0.88×10^{-2})
AptaMIP-4	3.58×10^{4} (± 0.94×10^{3})	4.77×10^{-1} (± 0.20×10^{-2})
AptaMIP-5	4.29×10^{4} (± 0.13×10^{3})	5.03×10^{-1} (± 0.12×10^{-2})
AptaMIP-6	7.47×10^{4} (± 0.45×10^{3})	1.75×10^{-1} (± 0.67×10^{-2})
AptaMIP-7	6.97×10^{4} (± 0.51×10^{3})	2.09×10^{-1} (± 0.84×10^{-2})
AptaMIP-8	5.34×10^{4} (± 0.38×10^{3})	2.26×10^{-1} (± 0.21×10^{-2})

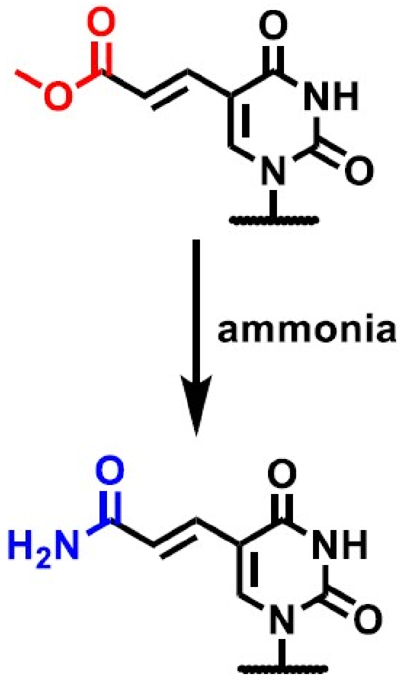

Figure S1: Amination of ester during synthesis

(a) AptaMIP-1 oligo

5’- ATC TAC GAA TTC ATC AGG GCT AAA GAG TGC AGA GTT ACT TAG -3’
(6 mods even spaced throughout)

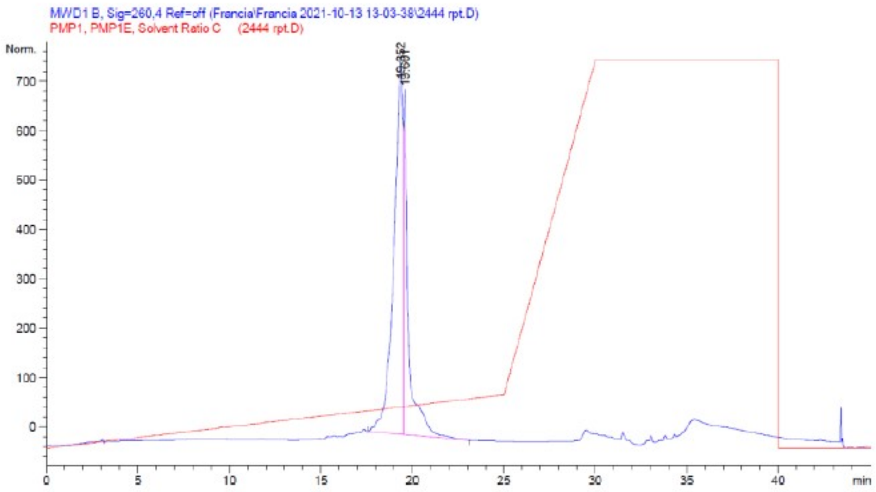

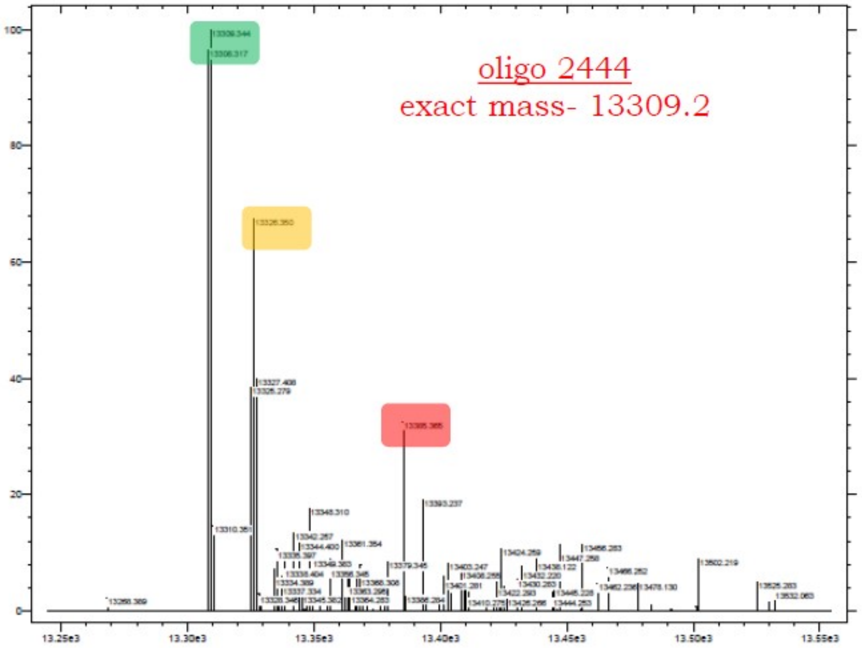

(b) AptaMIP-2 oligo

5'- ATC TAC GAA TTC ATC AGG GCT AAA GAG TGC AGA GTT ACT TAG -3'
(4 mods even spaced throughout)

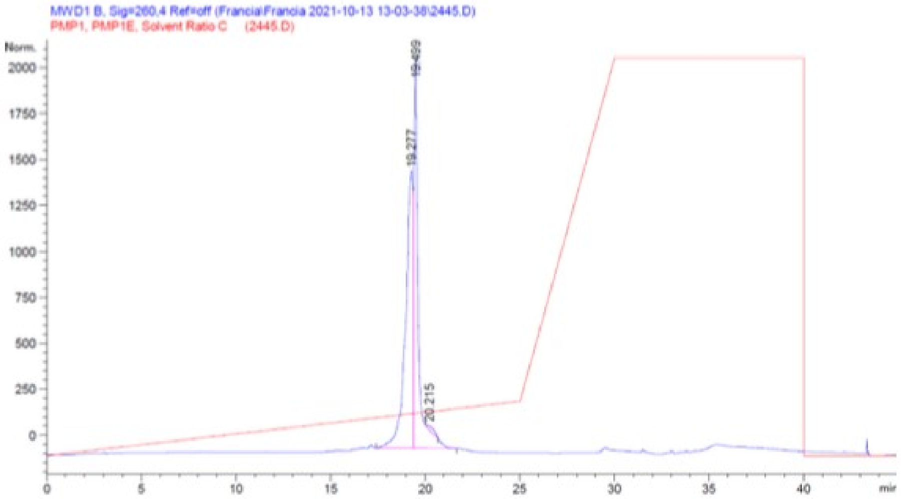

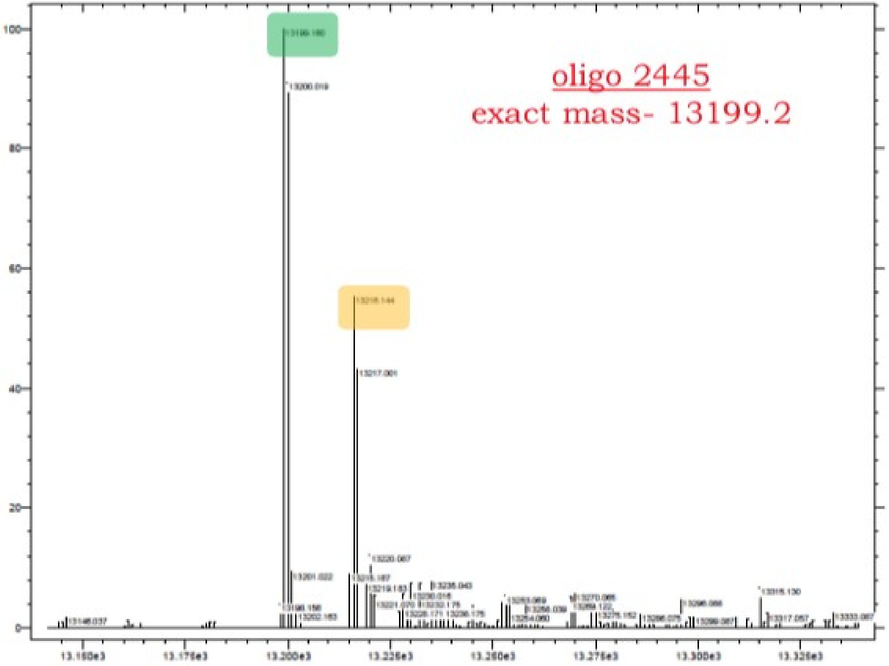

(c) AptaMIP-3 oligo

5'- ATC TAC GAA TTC ATC AGG GCT AAA GAG TGC AGA GTT ACT TAG -3'
(3 mods ends and centre)

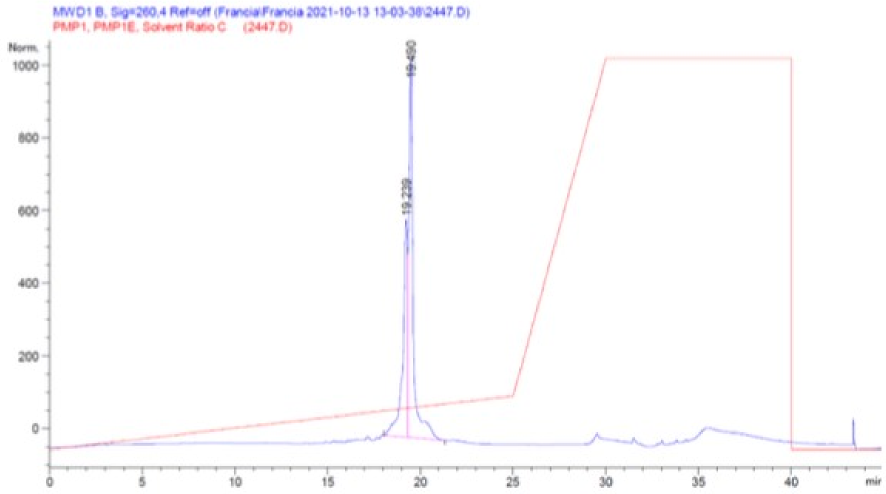

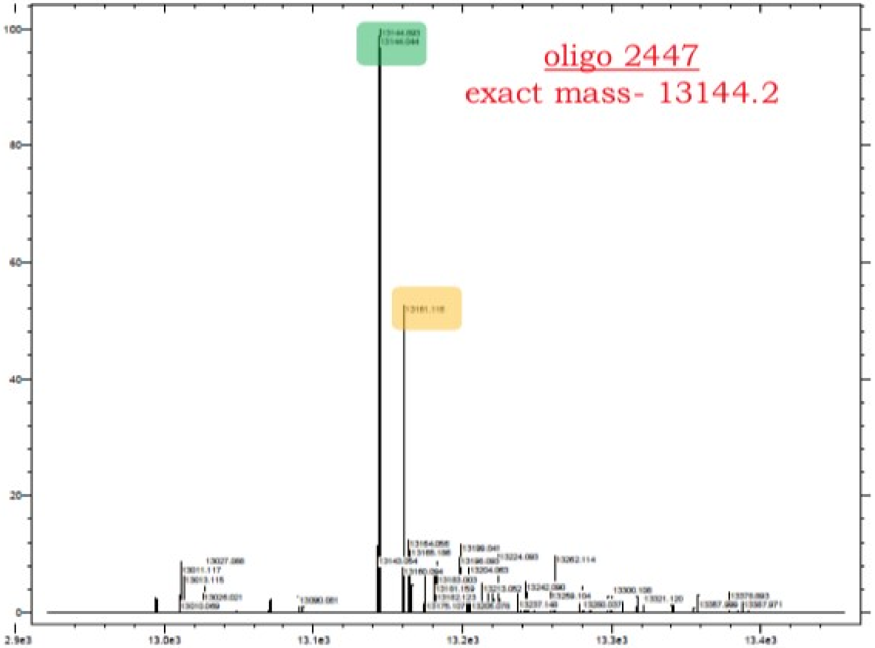

(d) AptaMIP-4 oligo

5'- ATC TAC GAA TTC ATC AGG GCT AAA GAG TGC AGA GTT ACT TAG -3' (3 mods centre of sequence)

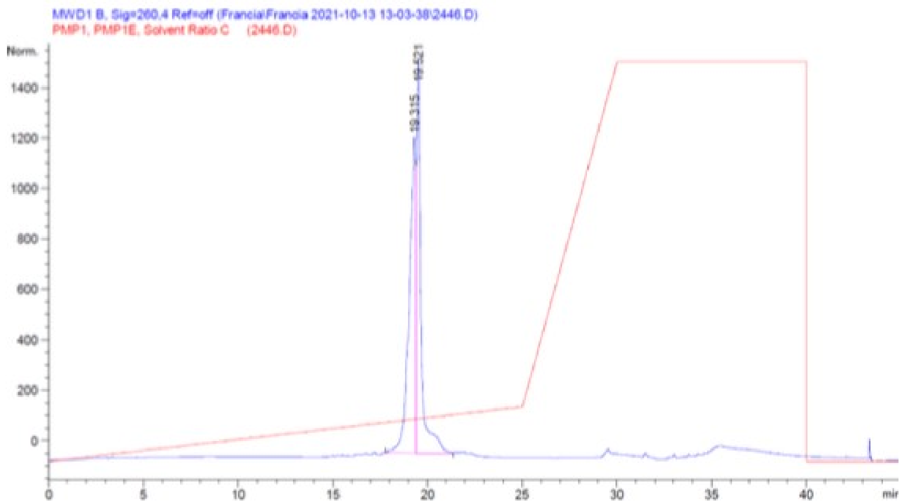

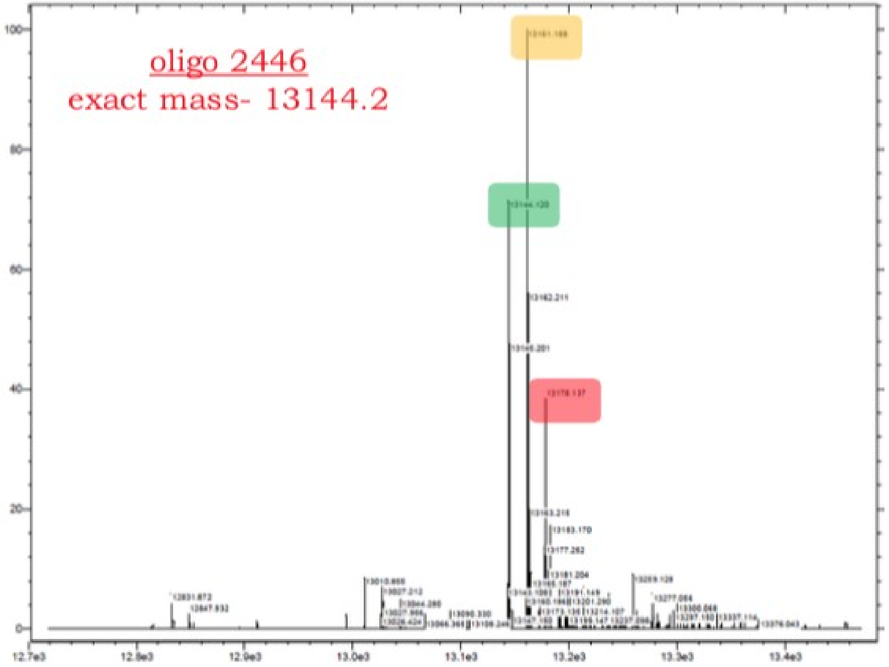

(e) AptaMIP-5 oligo

5’- ATC TAC GAA TTC ATC AGG GCT AAA GAG TGC AGA GTT ACT TAG -3’ (2 mods either end)

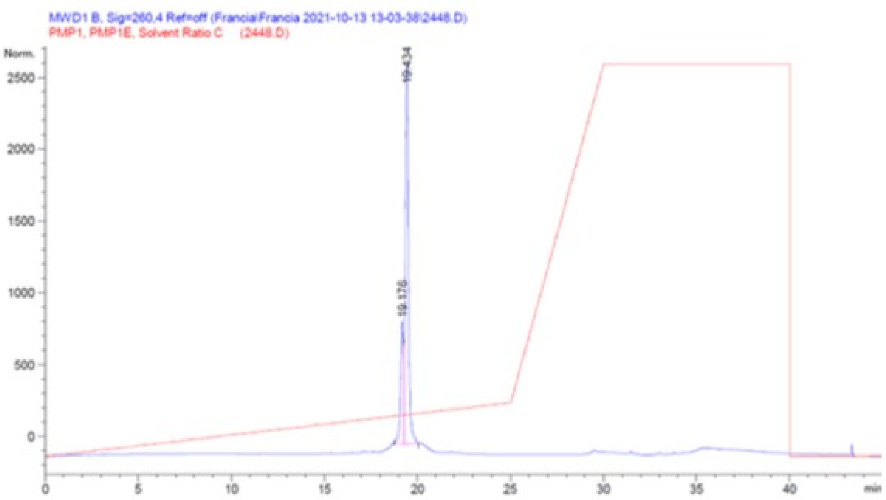

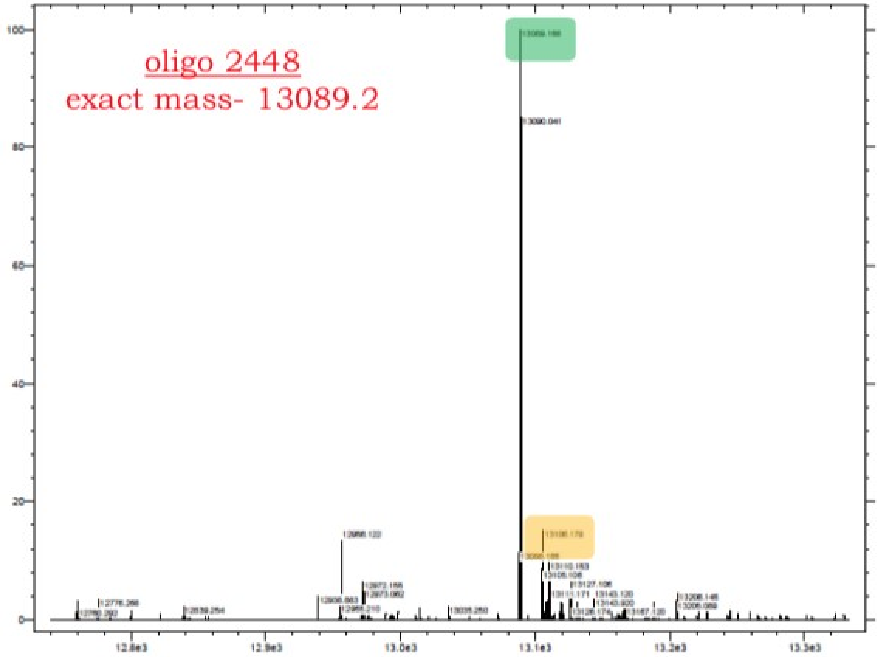

(f) AptaMIP-6 oligo

5’- ATC TAC GAA TTC ATC AGG GCT AAA GAG TGC AGA GTT ACT TAG -3’
(1 mod at 5’ end)

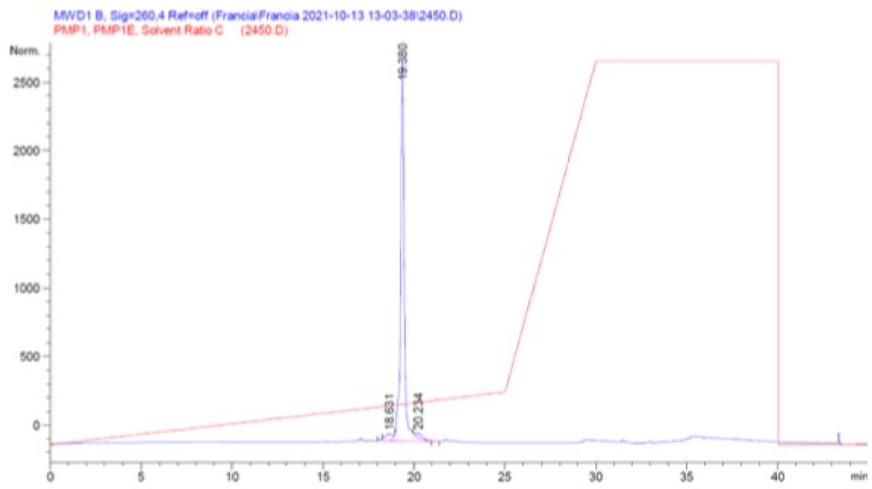

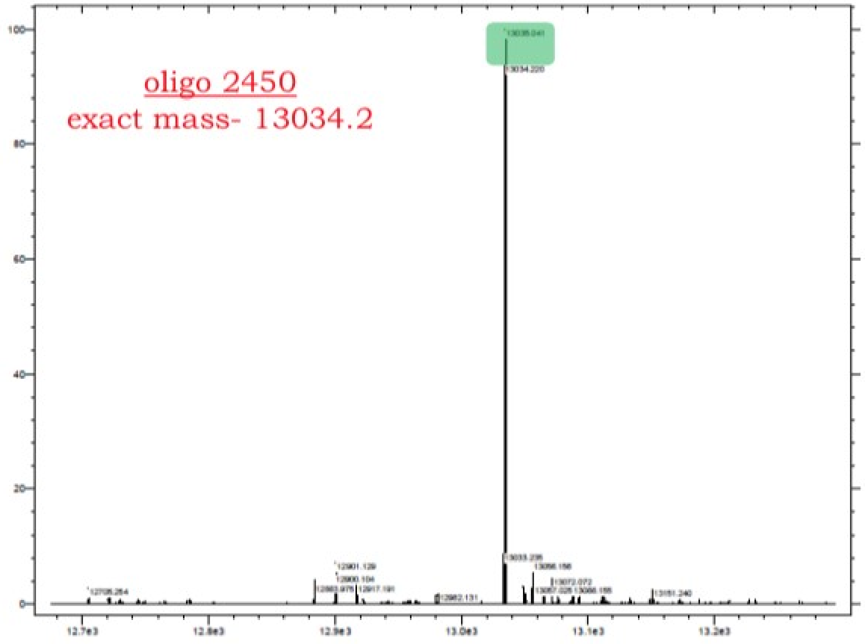

(g) AptaMIP-7 oligo

5’- ATC TAC GAA TTC ATC AGG GCT AAA GAG TGC AGA GTT ACT TAG -3’
(1 mod at 3’ end)

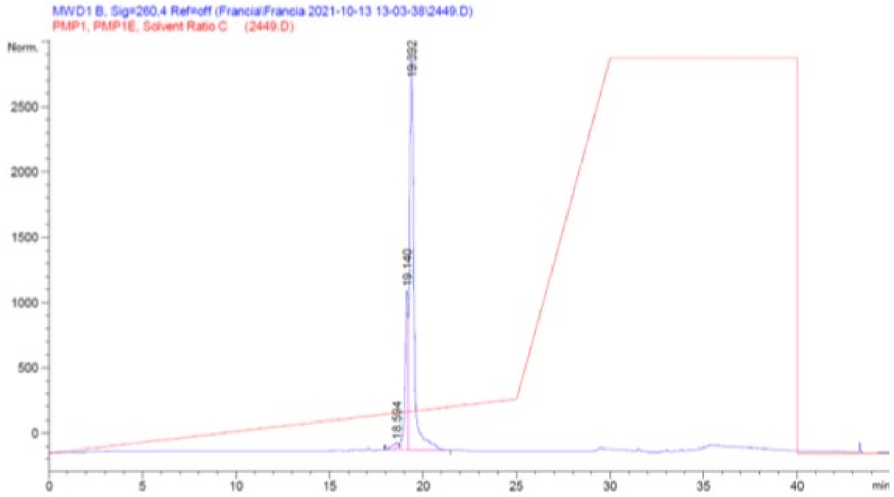

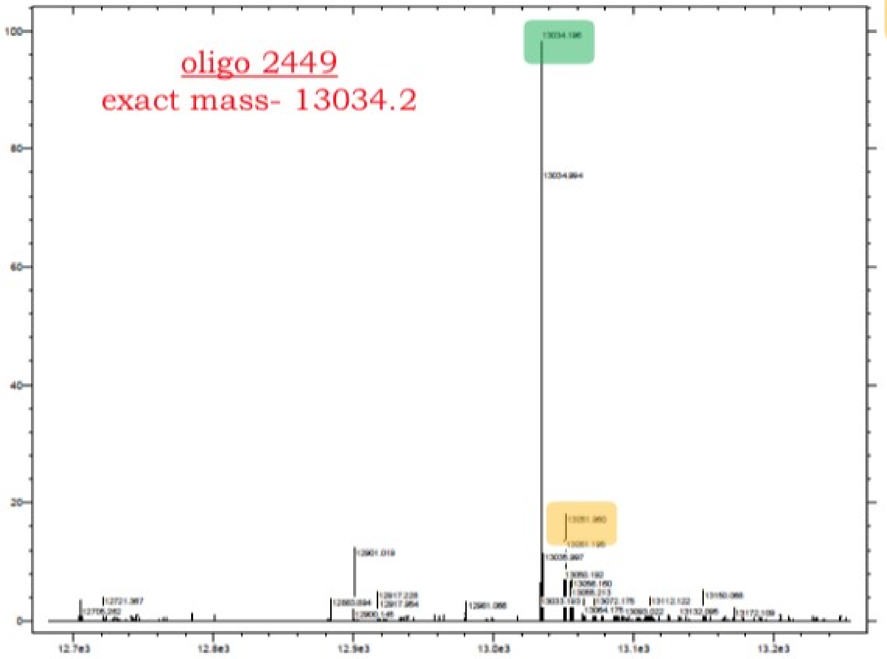

(h) AptaMIP-8 oligo

ATC TAC GAA TTC ATC AGG GCT AAA GAG TGC AGA GTT ACT TAG
(1 mod in centre)

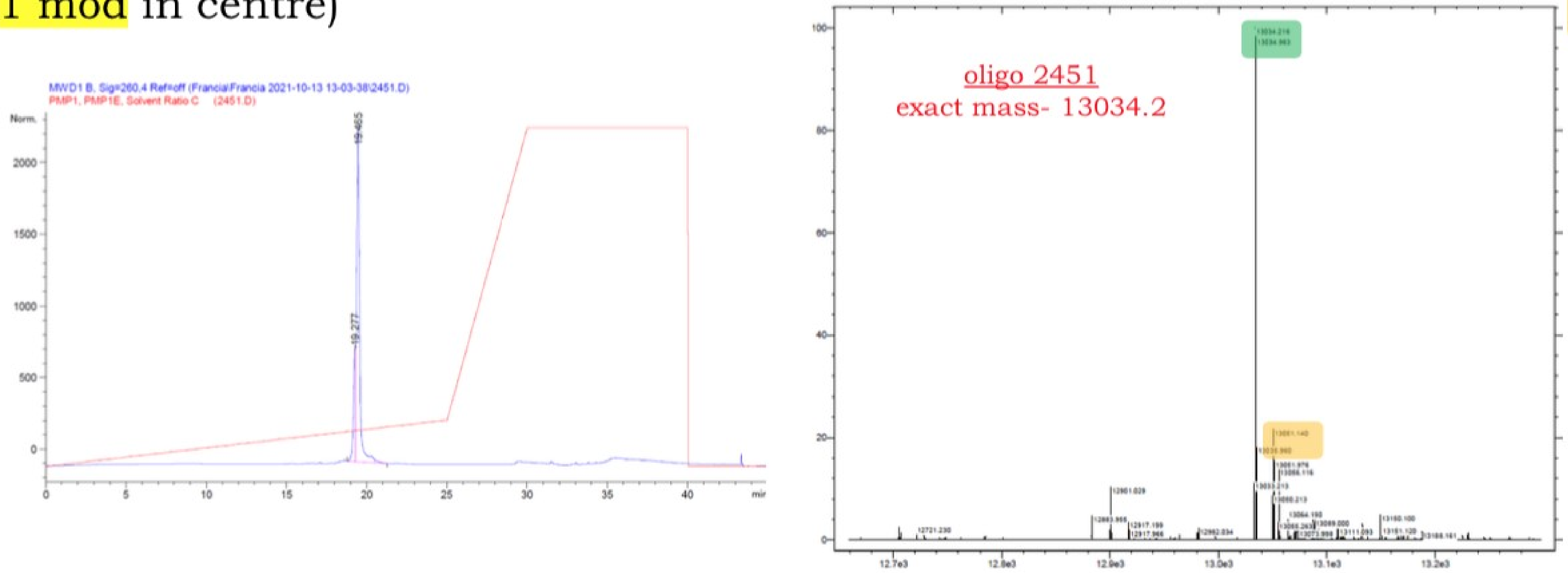

Figure S2: Reversed phase HPLC and ES-MS spectra of APTA-MIP oligonucleotide sequences. The MS spectra show highlighted peaks signals for the expected mass (full conversion to amide in green) and selected higher masses (one unconverted ester in orange and more than one unconverted ester in red). The authors are aware that the images are not the highest quality (due to the size of image and limitations of the supplementary docx. They are provided here as reference, hence the colour coding. Our lab runs an open data policy and, upon reasonable request, the data files can be requested via the corresponding author.

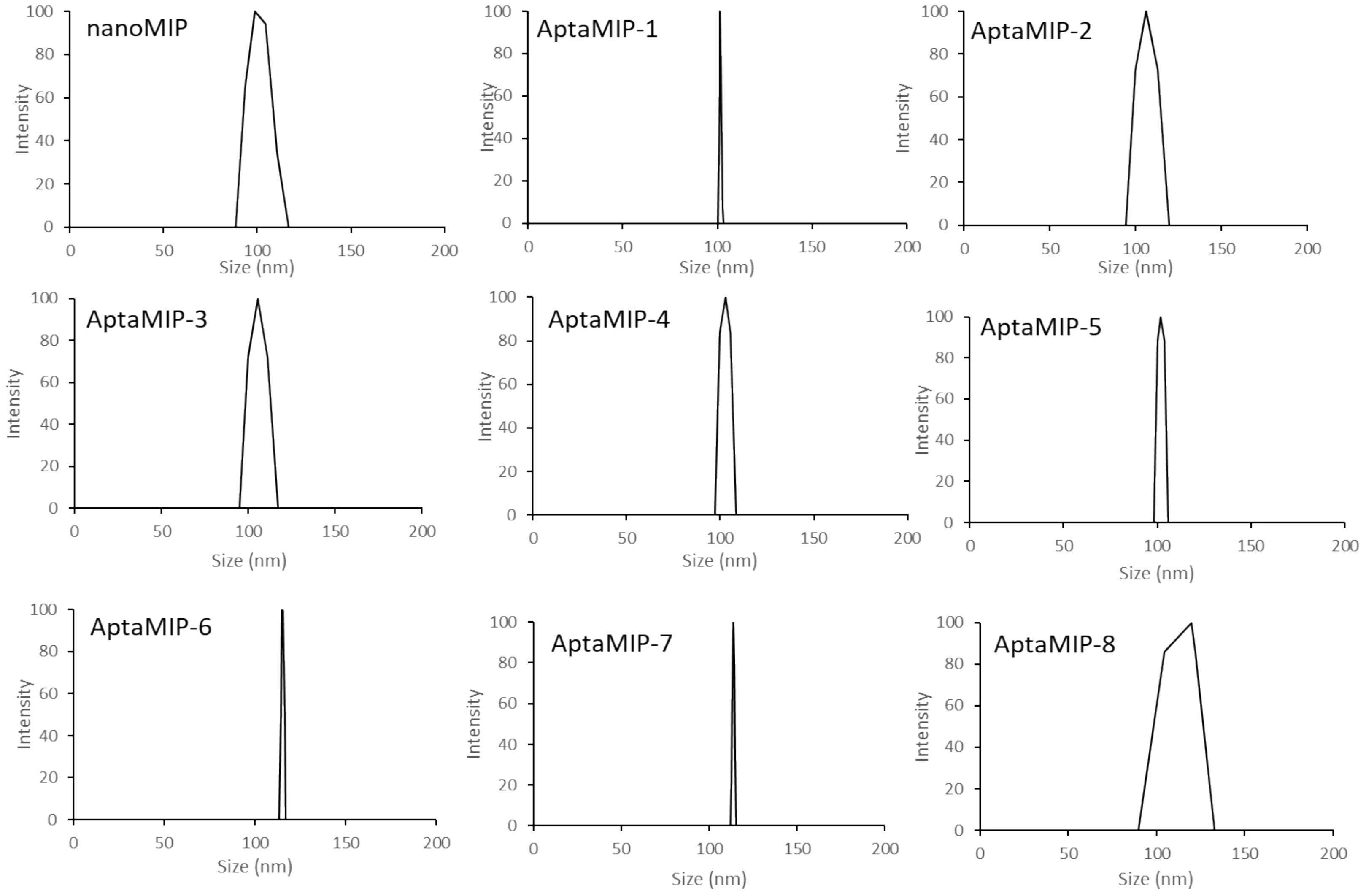

Figure S3a: DLS particle sizing for synthesised MIPs. Representative Curves. Experimental ran n=3 to acquire SD's.

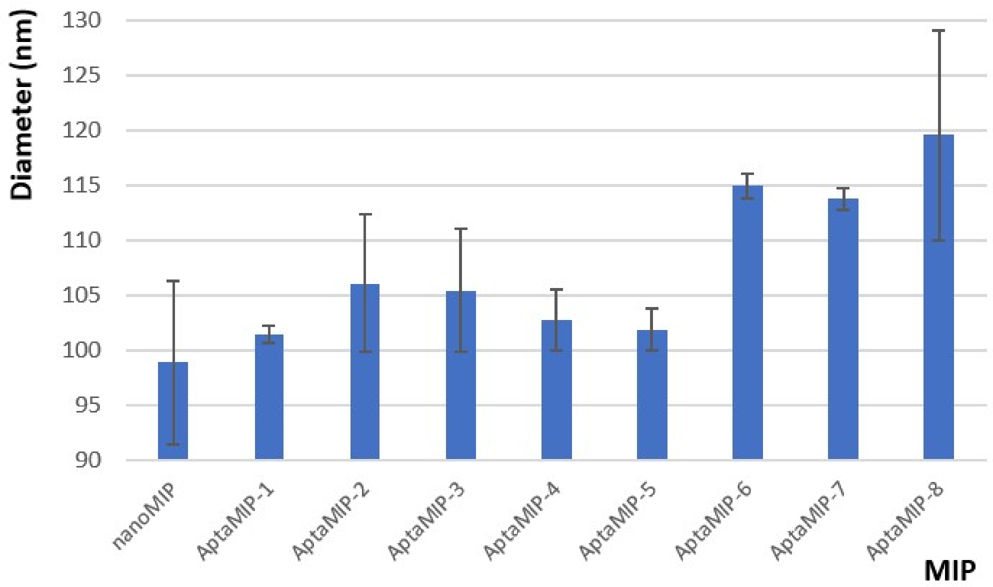

Figure S3b: DLS particle sizing for the synthesised MIPs. N = 5. Error bars highlight sample particle size distribution (Supplementary Figure S3). Truncated y-axis for clarity.

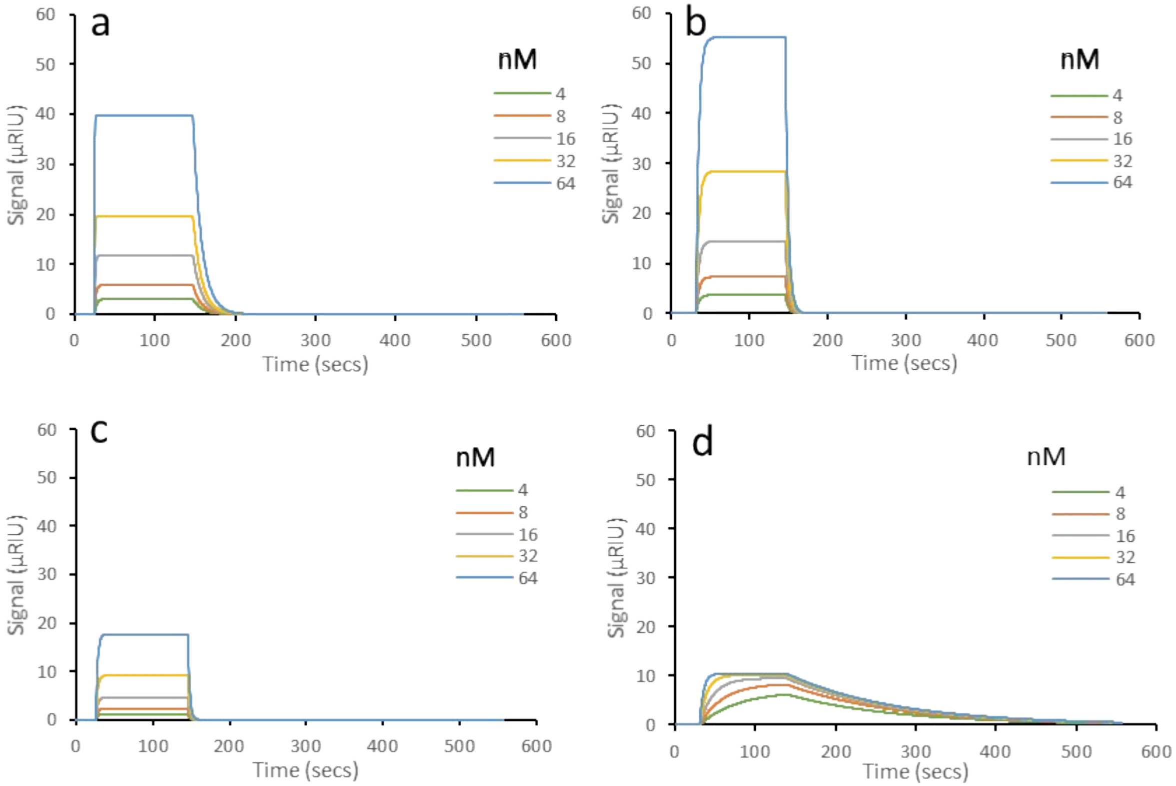

Figure S4a: Representative SPR sensorgrams of molecular interactions of imprinted nanoparticles immobilized on carboxymethyl dextran hydrogel coated Au chips. The SPR running buffer (PBST) was a phosphate buffered saline made at 10 mM, pH 7.4, supplemented with 0.01 % (v/v) Tween 20. Tween 20 is included to reduce non-specific binding during rebinding studies. Regeneration buffer was 10 mM Glycine-HCl at pH 2. All rebinding at 25 °C. All experiments in triplicate. Range of five concentrations (between 4 – 64 nM) for each protein. **(a)** AptaMIP-2 – Lysozyme. **(b)** AptaMIP-3 – Lysozyme. **(c)** AptaMIP-4 – Lysozyme. **(d)** AptaMIP-5 – Lysozyme. Related cross-reactivity studies (panels e – l) can be found in Figure S4.

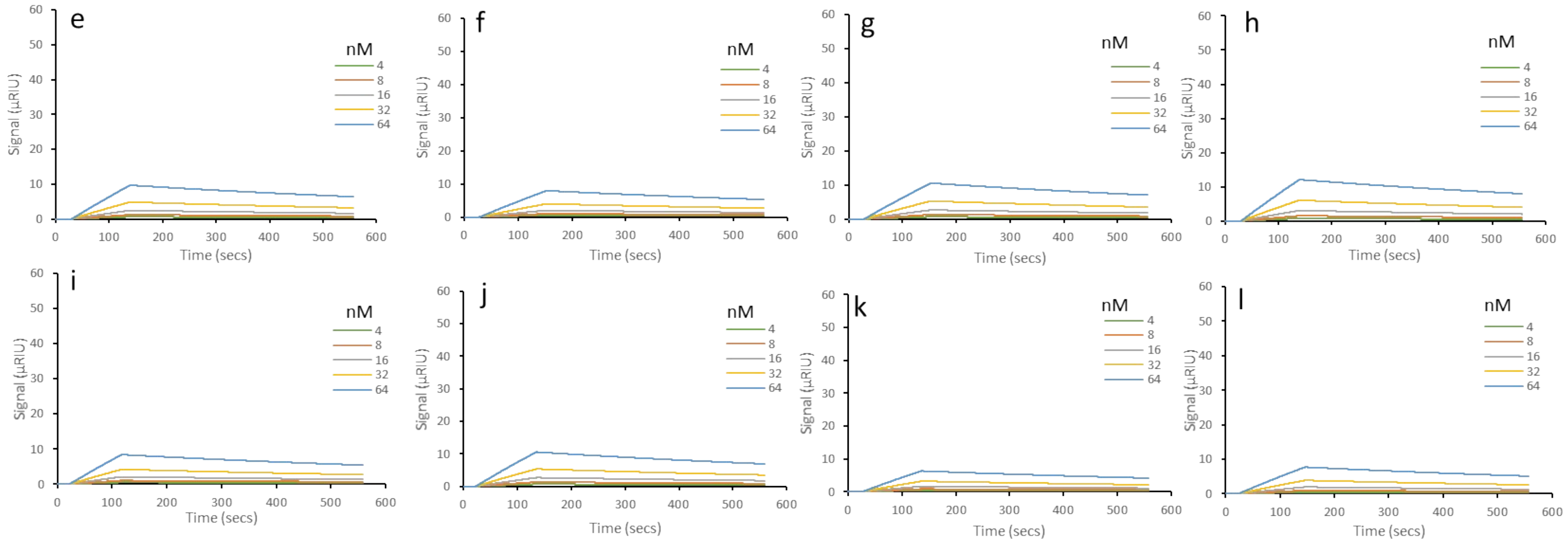

Figure S4b: Representative SPR sensorgrams of molecular interactions of imprinted nanoparticles immobilized on carboxymethyl dextran hydrogel coated Au chips. **Left Column:** AptaMIP-2. **Centre Left Column:** AptaMIP-3. **Centre Right Column:** AptaMIP-4. **Right Column:** AptaMIP-5. The SPR running buffer (PBST) was a phosphate buffered saline made at 10 mM, pH 7.4, supplemented with 0.01 % (v/v) Tween 20. Tween 20 is included to reduce non-specific binding during rebinding studies. Regeneration buffer was 10 mM Glycine-HCl at pH 2. All rebinding at 25 °C. All experiments in triplicate. Range of five concentrations (between 4 – 64 nM) for each protein. **(e)** AptaMIP-2 – Trypsin. **(f)** AptaMIP-3 – Trypsin. **(g)** AptaMIP-4 – Trypsin. **(h)** AptaMIP-5 – Trypsin. **(i)** AptaMIP-2 – BSA. **(j)** AptaMIP-3 – BSA. **(k)** AptaMIP-4 – BSA. **(l)** AptaMIP-5 – BSA. Related to Figure 5 in main manuscript.

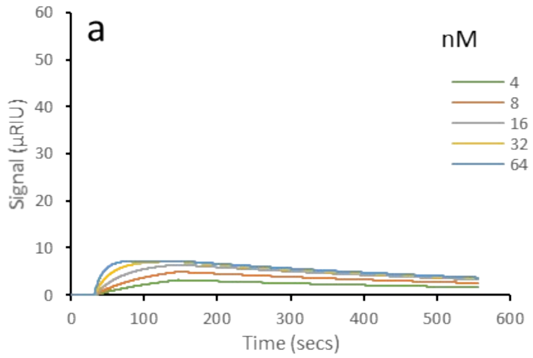

Figure S5a Representative SPR sensorgrams of molecular interactions of imprinted nanoparticles immobilized on carboxymethyl dextran hydrogel coated Au chips. The SPR running buffer (PBST) was a phosphate buffered saline made at 10 mM, pH 7.4, supplemented with 0.01 % (v/v) Tween 20. Tween 20 is included to reduce non-specific binding during rebinding studies. Regeneration buffer was 10 mM Glycine-HCl at pH 2. All rebinding at 25 °C. All experiments in triplicate. Range of five concentrations (between 4 – 64 nM) for each protein. **(a)** AptaMIP-6 – Lysozyme. **(b)** AptaMIP-7 – Lysozyme. **(c)** AptaMIP-8 – Lysozyme. Related cross-reactivity studies (panels d – i) can be found in Figure S5.

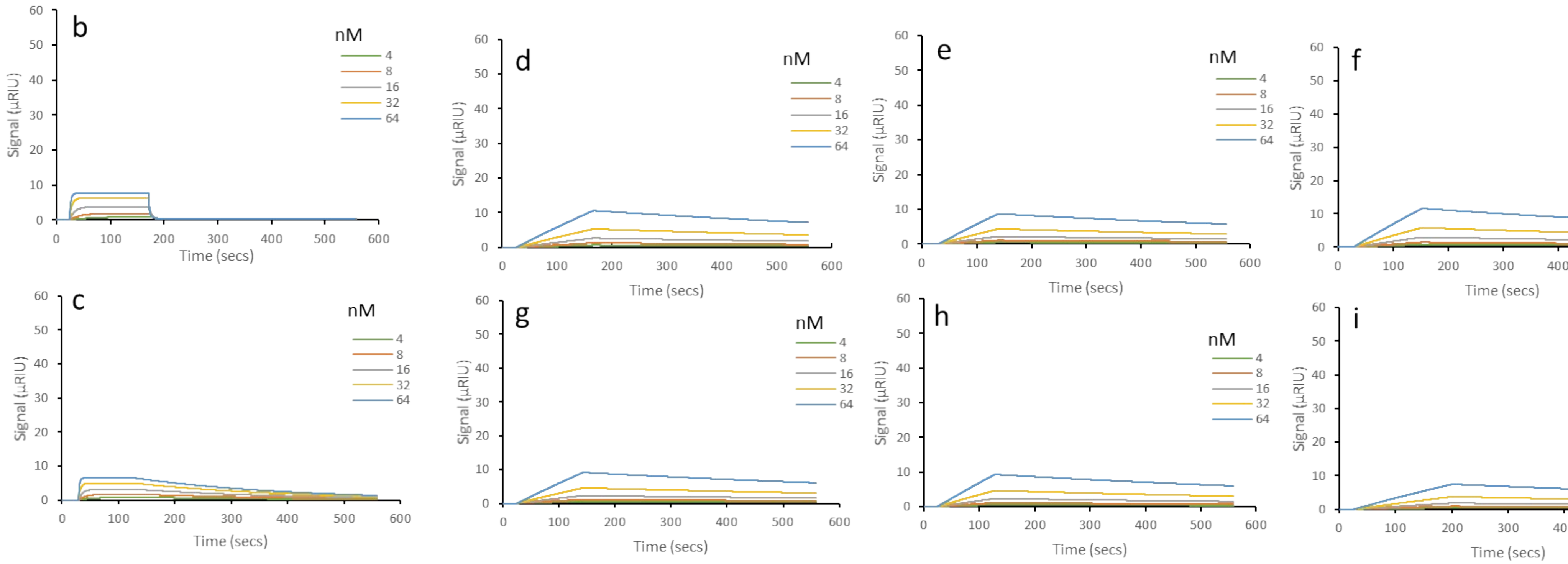

Figure S5b: Representative SPR sensorgrams of molecular interactions of imprinted nanoparticles immobilized on carboxymethyl dextran hydrogel coated Au chips. The SPR running buffer (PBST) was a phosphate buffered saline made at 10 mM, pH 7.4, supplemented with 0.01 % (v/v) Tween 20. Tween 20 is included to reduce non-specific binding during rebinding studies. Regeneration buffer was 10 mM Glycine-HCl at pH 2. All rebinding at 25 °C. All experiments in triplicate. Range of five concentrations (between 4 – 64 nM) for each protein. **(d)** AptaMIP-6 – Trypsin. **(e)** AptaMIP-7 – Trypsin. **(f)** AptaMIP-8 – Trypsin. **(g)** AptaMIP-6 – BSA. **(h)** AptaMIP-7 – BSA. **(i)** AptaMIP-8 – BSA. Related to Figure 6 in main manuscript.

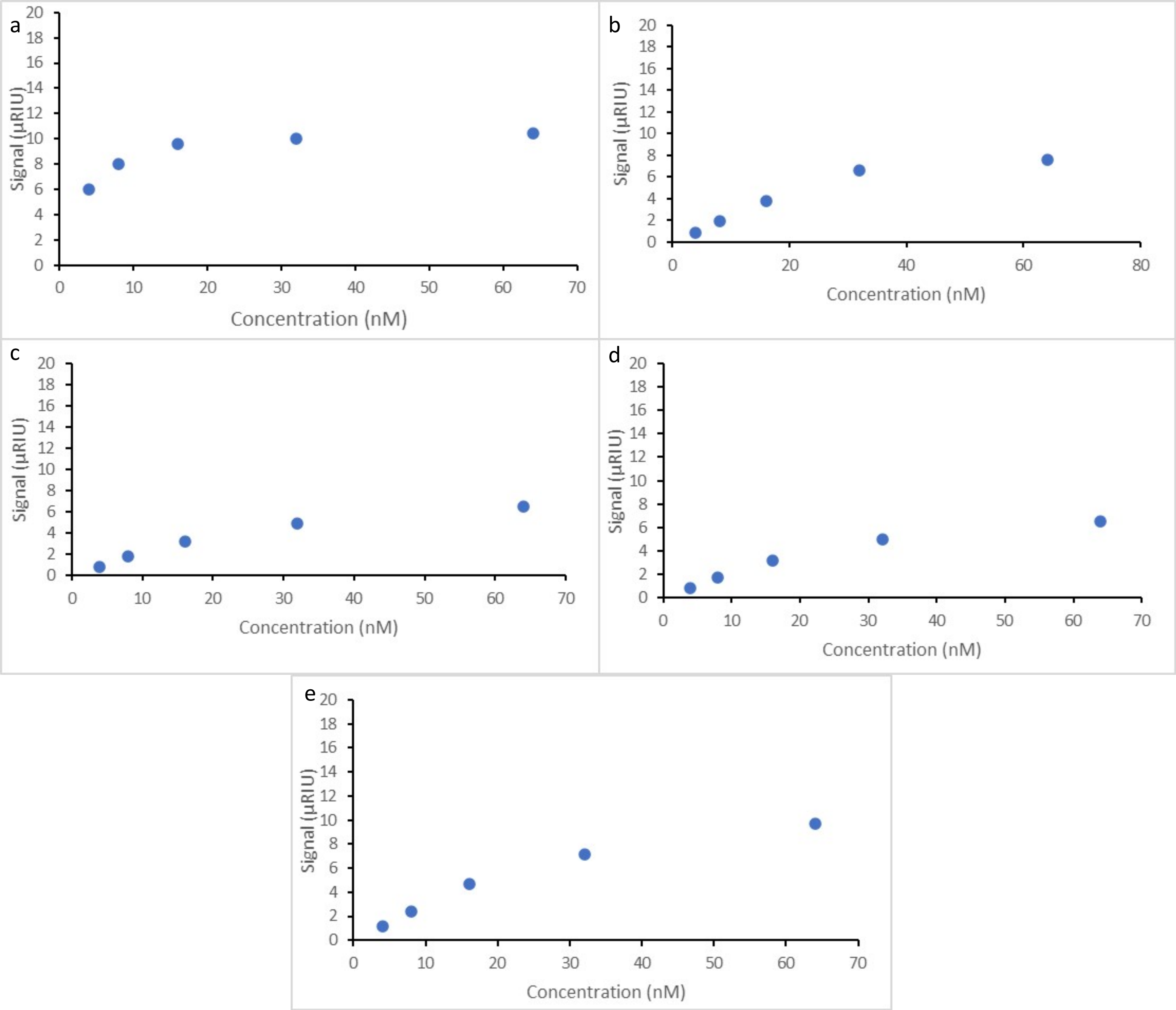

Figure S6: Elucidation of theoretic limit of detection for SPR sensor. Relative signal versus concentration. a) Lysozyme binding to AptaMIP 4, b) Lysozyme binding to AptaMIP 5, c) Lysozyme binding to AptaMIP 6, d) Lysozyme binding to AptaMIP 7, e) Lysozyme binding to AptaMIP 8.
